# Response to family violence in child health services (FRIDa): study protocol of a mixed-method study in the Stockholm region, Sweden

**DOI:** 10.1136/bmjopen-2025-115537

**Published:** 2026-07-22

**Authors:** Anna Fröjlinger, Lisa Blom, Kirsi Tiitinen Mekhail, Maria Lalouni, Lene Lindberg

**Affiliations:** 1 Department of Global Public Health, Karolinska Institutet, Stockholm, Sweden; 2 Department of Neurobiology, Care Sciences and Society, Karolinska Institutet, Stockholm, Sweden; 3 Department of Clinical Neuroscience, Karolinska Institute, Stockholm, Sweden; 4 Center for Epidemiology and Community Medicine, Region Stockholm, Stockholm, Sweden

**Keywords:** Gender-Based Violence, Child protection, Nursing research, Community child health

## Abstract

**Introduction:**

Growing up with family violence (FV) is potentially harmful both in a short-term and long-term perspective for the child. Early identification and support are therefore important. The purpose of this project (FRIDa) is to gain a deeper knowledge about the identification of and response to FV in the Swedish Child Health Services (CHS). Further, we would like to better understand how structure and organisation together with nurses’ perceptions may contribute to identification and response within the CHS.

**Methods and analysis:**

In this part of the FRIDa-project, we will use a mixed-method design to explore the pattern of identification of, and response to, FV. Approximately 90 000 CHS medical records over 12 months will be analysed with regression analysis to examine how FV identification and response vary by demographic factors. Furthermore, a qualitative interview study will gain information about the organisational barriers and facilitators and nurses’ professional experiences of working with FV within the CHS. The quantitative and qualitative results will be integrated in an explorative data analysis focusing on variations in the frequencies of posed questions, identification and response to FV supported by interview data.

**Ethics and dissemination:**

The study was approved by the Swedish Ethical Review Authority (Reference number 2025-03516-01). Results will be published with open access in international peer-review journals and communicated to national and regional CHS to contribute to the understanding of the long-term clinical work with FV within CHS. It will also add to the advancement of evidence-based practices for identification of, and response to, FV.

**Trial registration number:**

NCT07158983.

**Strengths and limitations of this study**

- A mixed-methods design integrates quantitative register data and qualitative interview data.
- The large register dataset of approximately 90 000 child health records provides high statistical power for quantitative analyses.
- The inclusion of two major child health service providers increases variation in organisational and socioeconomic contexts.
- The study is conducted within a single Swedish region, which may limit transferability to other regions and countries.
- Voluntary participation in the interview study may introduce selection bias among participating nurses.

## Introduction

Family violence (FV) is a serious global public health concern.^1^ The prevalence of FV varies depending on definition, how, when and where data has been collected.^2 3^ Children may experience FV through direct exposure (eg, physical, psychological or sexual abuse) or by witnessing violence between caregivers or other family members. While these forms of exposure often co-occur, they represent conceptually distinct experiences.^4^ Globally, a large proportion of children are exposed to FV.^2^ In Sweden, self-reported data from adolescents regarding experiences during childhood indicate that 12% and 13% have been subjected to psychological or physical abuse by a caregiver, respectively, and around 10% have witnessed violence between adults in the home.^5^

Short-term and long-term consequences for children experiencing FV have been described concerning physical health, cognitive, emotional and behavioural problems or disorders.^6^ Another identified consequence associated with exposure to FV during childhood is the risk of being a victim or perpetrator of FV in adulthood.^7^ The consequences may be similar whether the child has witnessed violence between adults or has been directly exposed to violence.^8^

Early identification of FV within child health services (CHS) is considered an important strategy for mitigating negative effects for children. In Sweden, CHS is a nurse-led, universal service offered free of charge to all children aged 0–5 years and their parents, reaching almost all families. All parents are offered an individual consultation with a CHS nurse during the child’s first 6 months, providing an important opportunity to ask about exposure to FV. The high coverage and repeated contacts over time make CHS a central arena for identifying FV.^9^

Whether detection of FV should be administered through questions on routine or by indication is not evident. Research so far has failed to demonstrate that routine questions about FV reduce the prevalence of FV.^10^ That may, however, be explained by lack of effective interventions, rather than by the identification process. WHO recommends that services with regular contact with women and children consider routine enquiry about FV, whereas services with less frequent contact may instead use targeted questions based on identified risk factors^11^

Few studies have examined how questions on exposure to violence posed to new fathers in the healthcare settings are working. A few Swedish studies from the CHS context report a lower share of fathers than mothers being asked about violence.^12 13^ To our knowledge, no international studies have investigated how fathers are offered the opportunity to answer questions about their own exposure to violence within a child health or parenting support context.

FV exists in all societies and cultures but there is an increased risk in areas with a higher degree of social vulnerability.^14^ Examining whether parents in areas with different socioeconomic conditions are equally likely to be asked about FV is therefore important. Social vulnerability at a societal level can be measured in several ways. One such measure used within CHS is Care Need Index (CNI), a socioeconomic index used to estimate healthcare needs within an area.^15^ It is based on factors such as single parenthood, unemployment, low educational level and proportion of foreign-born residents in the area. A higher CNI indicates a greater need for support and resources in primary healthcare.

Maintaining routines for asking about violence over time can be difficult, as these practices are often influenced by factors such as workload, staff turnover and access to training. Even routines that are initially well implemented may diminish or become less consistently applied over time.^16^ These organisational factors may also influence healthcare professionals’ confidence in addressing violence, including their preparedness to act when violence is identified. Previous research suggests that a prerequisite for healthcare professionals to ask about exposure to violence is to feel confident about actions and steps to follow if violence is identified.^17^ In Swedish CHS, existing research has focused on the implementation phase of such routines.^12 13 17^ Less is known about how these practices are sustained over time and how nurses experience working with them in everyday clinical practice.

The present study addresses this gap by examining the experiences of CHS nurses who have worked with this approach for several years, providing insights into long-term practice rather than initial adoption. Furthermore, the approach in this study includes asking both mothers and fathers in the same family about their own exposure to violence, which is uncommon in previous research. This perspective is important for understanding how routine questions can support violence prevention and parenting support in a more inclusive way.

To sum up, exposure to FV among children is a severe public health problem that could be reduced by early identification and response in CHS. More research is needed to examine the opportunities of mothers and fathers and parents in different socioeconomic areas to be asked about violence during CHS encounters. There is also a need for increased knowledge of the support CHS nurses require to ask about violence and respond when violence is identified, particularly from a long-term perspective.

## Methods and analysis

### Aim

The aim of this project is to gain a deeper knowledge on how FV is identified and responses to FV within the Swedish CHS. Further, we aim to understand how structure and organisation together with nurses’ perceptions may contribute to identification of and response to FV within the CHS.

### Research questions

The specific research questions are:

- To what extent are questions about FV posed to mothers and fathers, respectively, in CHS?
- To what extent is ongoing FV identified among parents that have been asked about it?
- To what extent have reports of concern been made to social services when FV is identified?
- To what extent are structural factors (eg, CNI) and organisational conditions (eg, professional education and supervision) associated with nurses’ likelihood of inquiring about FV and responding when FV is identified within CHS?
- What barriers and facilitators do nurses within CHS perceive when asking about FV?
- What barriers and facilitators do nurses within CHS perceive in providing support to families affected by identified FV?

### Design

The study will use a mixed-method design including both quantitative and qualitative approaches. In the quantitative part of the study, data from CHS medical records will be used to identify the frequency of questions posed on routine and based on indication and reports of FV within the CHS. Data from clinical managers are used to map the organisational preconditions of the CHS clinics. The qualitative part of the study consists of interviews with nurses to explore their experiences of identifying and responding to identified FV. The combination of methods will support the understanding of the situation around working with identifying FV within CHS. The study protocol was developed according to the Good Reporting of a Mixed Methods Study (GRAMMS). GRAMMS is used as a checklist to ensure that relevant information is included in the study protocol and it will also be used in the reporting of the results.^18^

### Study setting

The study is set in the Stockholm region in Sweden and involves two of the largest caregivers providing CHS in Stockholm region—Stockholm Health Care Services and Capio. Together they account for about half of the children enrolled at CHS clinics in the region. The Stockholm region comprises about 20% of all children residing in Sweden.

FRIDa is a research project aimed at supporting CHS in identifying and responding to FV through structured routines and staff training.^19^ Based on the FRIDa model, routine questions about FV have been implemented in the CHS in the Stockholm region. For the mother, this usually takes place during an individual visit when the child is 6–8 weeks and for the father at an individual visit when the child is 3–5 months. Three questions are asked, relating to (1) prior exposure to FV for the parent, (2) ongoing exposure to FV for the parent and (3) ongoing exposure to FV for the child. Questions on exposure to violence should also be asked at any time based on indication, whenever there is a suspicion of violence.

### Quantitative part

#### Objectives

The objective of the quantitative part of the study is to examine how questions about FV are asked within CHS in the Stockholm region, and how healthcare professionals respond when violence is identified. A further objective is to examine how these practices are associated with organisational and contextual factors at the CHS clinic level.

#### Data sources

The quantitative part will draw on two complementary data sources:

#### Registry-based data from CHS medical records

##### Participants

We will make use of registry data from medical records of children enrolled in the CHS during the period 30 June 2024 to 30 June 2025. Approximately 90 000 children are enrolled in the CHS provided by the two included healthcare providers. The included CHS clinics are based in areas with different socioeconomic characteristics (according to CNI) located in the city, suburbs and surrounding municipalities in the Stockholm region.

##### Measurements and data collection

De-identified medical records from CHS will be compiled with support from administrators at the two care providers. There are specific variable names in the medical records for questions about exposure to violence, reports of concern to social services, and whether the mother or father had an individual visit with the CHS nurse.

The data that will be retrieved from the CHS medical records are: date for the visit; age of the child; type of contact; information about whether an individual visit has taken place for the parent (yes/no); whether a question on exposure to violence has been asked (yes/no) and the parents’ answer; notes about exposure to violence for the child (yes/no); reports of concern to social services (yes/no); and reason for reports of concern to social services (when a report is made).

Since questions about violence should be asked whenever there is a suspicion of violence, all medical records from the CHS of the two care providers are eligible for inclusion in the data collection. The total number of enrolled children will be used as the denominator when calculating frequencies. Information on FV assessment will be extracted from medical records where documentation of (1) an individual parental consultation, (2) questions about violence or (3) a report of concern is present during the study period.

#### Organisational characteristics of CHS clinics

##### Sampling and recruitment

All unit managers and operations managers at the participating CHS clinics will be contacted.

##### Data collection

An electronic questionnaire with questions about organisational characteristics of the included CHS clinics will be distributed via a secure web platform (see online supplemental file 1). The questions cover information on the number of nurses employed, their percentage of full-time employment, years of experience in CHS, number of enrolled children and how many of the nurses that have participated in training about FV. The questionnaire also includes questions on staff turnover in the latest year. It is estimated to take about 15 min to complete the questionnaire. The CNI for each CHS clinic is publicly available and will be retrieved from Region Stockholm.

10.1136/bmjopen-2025-115537.supp1 Supplementary data

##### Informed consent

An information letter will be distributed to all unit managers and operations managers of the included CHS clinics. The information letter includes information about the voluntariness to complete the questionnaire, that all information will be reported on a group level without possibilities to identify specific CHS clinics or nurses and that the data collected will only be available to the researchers involved and stored securely at Karolinska Institutet.

##### Data integration

Data from medical records on FV assessment will be linked to corresponding clinic-level variables, including CNI and organisational characteristics, to examine how contextual factors may be associated with assessment practices.

##### Analysis

Descriptive statistics will be used to calculate the frequency of (1) questions about FV posed to parents, (2) identification of ongoing violence and (3) reports of concern following identification. Differences between mothers and fathers in being asked about violence will be examined using appropriate statistical tests depending on the data distribution. Regression analyses will be conducted to explore associations between questions about violence, CNI and organisational and clinic-level variables. All analyses will be conducted using the statistical software IBM SPSS Statistics for Windows, V.31.0 (IBM).

### The qualitative part

#### Objectives

The objective of the qualitative part of the study is to gain insight into nurses’ perspectives on, and experiences from, working with questions about FV in CHS and how it is embedded and sustained in the routine practice. An additional objective is to better understand how they respond when FV is identified.

#### Sampling and recruitment

Based on the results of the quantitative part, a selection of 10–15 nurses will be approached and asked for participation in semistructured individual interviews. The selection will consist of nurses working at CHS clinics with different CNI and with high and low frequencies of parents being asked about FV according to the results from the quantitative part.

#### Data collection

Semistructured individual interviews will be conducted focusing on barriers and facilitating factors to pose questions on exposure to violence and to respond when violence exposure is identified. The interview guide (see online supplemental file 2) is informed by the Consolidated Framework for Implementation Research that can be used to support the understanding of factors affecting the implementation of evidence-based methods and interventions.^16^ The guide includes questions addressing experiences of asking about violence, perceived clarity of guidelines, organisational support and conditions, collaboration with external actors, as well as individual confidence and preparedness to ask about and respond to violence. The interviews will be conducted face-to-face or digitally via Zoom or Teams after informed consent has been signed. The interviews will be audio-recorded and thereafter transcribed verbatim.

10.1136/bmjopen-2025-115537.supp2 Supplementary data

#### Informed consent

Written informed consent will be obtained from the nurses prior to participating in the interviews. The nurses will be informed that participation is voluntary and that data will be presented in a way minimising the risk of identifying individual participants. Nurses participating in the interviews will also have access to support from a psychologist from the study team.

#### Analysis

The interviews will be analysed using reflexive thematic analysis.^20^ The analysis will involve an iterative and recursive process, moving back and forth between reading the transcripts, coding the data, and developing themes. The researchers will read the transcripts and contribute to the analytic process through ongoing discussion. The focus of the analysis will be on exploring variations in nurses’ experiences of asking about, identifying, and responding to FV within CHS.

### Integration of the results from the quantitative and the qualitative parts

Results from the two parts will be integrated in an explorative data analysis with a mixed-method approach.^21^ The focus on the analysis will be to explore variations in the frequency of questions posed, identification and response to FV supported by interview data.

### Study timeline

Ethical approval was sought and granted in May 2025. The caregivers were approached and requested for patient and organisational data during October/November 2025. Data management and analysis of the register data was initiated in February 2026 and will be finalised in November 2026. Nurses at selected CHS clinics will be contacted with written information and invitation to participate in interviews in January 2027. Interviews are planned to be conducted in March–June 2027. Data management and analysis of the interview data will be conducted in March–December 2027. The integration of the results of the material from the registry review and the interviews will be compiled and presented in January–June 2028. The timeline of the study is described in online supplemental file 3 table 1.

10.1136/bmjopen-2025-115537.supp3 Supplementary data

### Ethics and dissemination

The study was approved by the Swedish Ethical Review Authority (Reference number 2025-03516-01). Nurses will provide written informed consent before participating in the interviews. All results will be presented at a group level. The final data set will be handled in accordance with the guidelines of Karolinska Institutet. The variable list and the information letters are available from the corresponding author on request.

The results of the study will be published with open access in peer-reviewed journals and presented to regional and national CHS.

### Patient and public involvement

Patients and the public were not involved in the design, conduct, reporting or dissemination plans of this research. However, the results will be shared with CHS staff to inform practice.

## Discussion

This protocol describes a mixed-methods study designed to gain a deeper knowledge on identification of, and response to, FV within the Swedish CHS. Considering the serious health consequences that violence can cause, it is important that healthcare introduces evidence-based methods that facilitate identification and ensure equitable opportunities for support for victims of violence.

There are several practical and operational issues to discuss and reflect on in the upcoming study. One relates to the two outcomes identification of exposure to violence for and reports of concern to social services. Both outcomes are rare events which can make comparisons difficult, as reported in a previous study within the research group.^19^ The large material in this study is expected to cater for better comparisons.

Another issue concerns the selection of CHS clinics. Only two care providers of CHS are included due to practical and logistical reasons. However, the two care providers together account for a large share of the CHS clinics in the uptake area and include clinics located in areas with varying socioeconomic conditions and are expected to provide a representative sample of the CHS clinics in the region.

Several considerations related to the selection of nurses and the interviews should also be lifted. The selection of nurses is made with the purpose of providing information from a variation of CHS clinics with different CNI and with low and high frequencies of parents that have been asked about exposure to violence. The nurses are selected based on the level of the CHS clinic instead of the individual nurses’ levels, to decrease the risk of nurses feeling exposed. A downside of that is that we will not be able to control the selection of the individual nurses at a specific clinic, where nurses with a more positive perspective on asking questions around violence might be more willing to participate in the interviews. To mitigate this, the study emphasises that interviews focus on barriers and facilitators and that confidentiality will be strictly maintained. Another concern is the choice of interviewer which may affect how comfortable nurses feel about sharing their experiences and perspectives. We will strive to select an interviewer that has no, or limited, connection to the day-to-day work of the nurses.

The study is expected to support the possibilities for children’s and parents’ equal opportunities for support via the CHS in case of FV. This is expected to be achieved by increasing knowledge about potential differences in how questions on violence are posed to parents related to gender, area level socioeconomic background and different possibilities, both actual preconditions and experienced opportunities, for nurses to comply with the method implemented. A strength of the study is its focus on nurses who have worked with the method for several years, offering insights into sustainability and normalisation of the approach beyond the initial implementation phase. This long-term perspective is important for understanding not only feasibility but also how the method becomes embedded in routine practice. The findings may inform strategies for organisational support and training, ensuring that CHS nurses have the necessary conditions to maintain this practice over time. This could strengthen the capacity of CHS to identify FV early and provide equal opportunities for support to all parents, contributing to violence prevention and safer environments for children.

## Supplementary Material

Reviewer comments

Author's manuscript

## References

[1] Krug EG, Mercy JA, Dahlberg LL, et al. The world report on violence and health. Lancet 2002;360:1083–8. 10.1016/S0140-6736(02)11133-0 12384003

[2] UNICEF. A familiar face: violence in the lives of children and adolescents. New York, 2017.

[3] World Health Organization. Violence against women prevalence estimates, 2018: global, regional and national prevalence estimates for intimate partner violence against women and global and regional prevalence estimates for non-partner sexual violence against women. Geneva World Health Organization; 2021.

[4] Howell KH, Taylor NR, Thomsen KN, et al. Global perspectives on family violence: prevalence and effects on children across cultural contexts. In: Psychological perspectives on understanding and addressing violence against children. 2022.

[5] Jernbro C, Landberg Å, Thulin J. Våld mot barn 2022: en nationell kartläggning. Report No.: 91-86759-57-4978-91-86759-57-5. Stockholm, 2023.

[6] Howell KH, Barnes SE, Miller LE, et al. Developmental variations in the impact of intimate partner violence exposure during childhood. J Inj Violence Res 2016;8:43–57. 10.5249/jivr.v8i1.663 26804945 PMC4729333

[7] Ehrensaft MK, Langhinrichsen-Rohling J. Intergenerational transmission of intimate partner violence: summary and current research on processes of transmission. In: Handbook of interpersonal violence and abuse across the lifespan. 2022: 2485–509.

[8] McTavish JR, MacGregor JCD, Wathen CN, et al. Children’s exposure to intimate partner violence: an overview. Int Rev Psychiatry 2016;28:504–18. 10.1080/09540261.2016.1205001 27414209

[9] Rikshandboken i barnhälsovård [Swedish National Handbook for Child Health services. Hälsobesök i alla åldrar: Inera AB. 2024. Available: https://www.rikshandboken-bhv.se/halsobesok/halsobesok-i-alla-aldrar/

[10] O’Doherty L, Hegarty K, Ramsay J, et al. Screening women for intimate partner violence in healthcare settings. Cochrane Database Syst Rev 2015;2015:CD007007. 10.1002/14651858.CD007007.pub3 23633338

[11] World Health Organization. Responding to intimate partner violence and sexual violence against women. WHO clinical and policy guidelines. Report no.: 9789240691797. Geneva World Health Organization; 2013. 24354041

[12] Nimborg J, Lindskog U, Nordgren L, et al. Routine conversations about violence conducted in Swedish child health services—A mixed methods study of nurses’ experiences. Acta Paediatr 2023;112:442–51. 10.1111/apa.16355 35398911 PMC10084205

[13] Engström M, Lindqvist S, Janson S, et al. Validation of the Swedish version of the safe environment for every kid (SEEK) parent screening questionnaire. BMC Public Health 2023;23:1989. 10.1186/s12889-023-16792-4 37828478 PMC10571478

[14] Capaldi DM, Knoble NB, Shortt JW, et al. A Systematic Review of Risk Factors for Intimate Partner Violence. Partner Abuse 2012;3:231–80. 10.1891/1946-6560.3.2.231 22754606 PMC3384540

[15] Sundquist K, Malmström M, Johansson SE, et al. Care Need Index, a useful tool for the distribution of primary health care resources. J Epidemiol Community Health 2003;57:347–52. 10.1136/jech.57.5.347 12700218 PMC1732439

[16] Damschroder LJ, Reardon CM, Widerquist MAO, et al. The updated Consolidated Framework for Implementation Research based on user feedback. Implement Sci 2022;17:75. 10.1186/s13012-022-01245-0 36309746 PMC9617234

[17] Anderzen-Carlsson A, Gillå C, Lind M, et al. Child healthcare nurses’ experiences of asking new mothers about intimate partner violence. J Clin Nurs 2018;27:2752–62. 10.1111/jocn.14242 29274181

[18] O’cathain A, Murphy E, Nicholl J. The Quality of Mixed Methods Studies in Health Services Research. J Health Serv Res Policy 2008;13:92–8. 10.1258/jhsrp.2007.007074 18416914

[19] Fröjlinger A, Lalouni M, Blom L, et al. n.d. Identification and response to family violence within Swedish Child Health Services: a pragmatic randomized controlled trial (manuscript submitted).

[20] Braun V, Clarke V. Conceptual and design thinking for thematic analysis. Qualitative Psychology 2022;9:3–26. 10.1037/qup0000196

[21] Fetters MD, Curry LA, Creswell JW. Achieving integration in mixed methods designs-principles and practices. Health Serv Res 2013;48:2134–56. 10.1111/1475-6773.12117 24279835 PMC4097839

