# Supplementary material for: Response to family violence in child health services (FRIDa): study protocol of a mixed-method study in the Stockholm region, Sweden
Source: BMJ Open. 2026 Jul 22;16(7):e115537. doi: 10.1136/bmjopen-2025-115537 (PMC13410707; doi:10.1136/bmjopen-2025-115537)
Supplement: Supplementary data [file bmjopen-16-7-s001.pdf]

Questionnaire for Child Health Centre Manager

- What is the name of the child health centre you manage?
- How many child health nurses were employed at the child health centre on July 1, 2024?
- How many child health nurses were employed at the child health centre on July 1, 2025?
- What percentage of the unit's child health nurses' working time is spent on child health services?
- How many child health nurses were employed during the period July 1, 2024 – July 1, 2025?
- How many child health nurses left during the period July 1, 2024 – July 1, 2025?
- For how many years have the unit's child health nurses worked in child health services?
- How many of the unit's child health nurses have participated in training on family violence?
