# Supplementary material for: Response to family violence in child health services (FRIDa): study protocol of a mixed-method study in the Stockholm region, Sweden
Source: BMJ Open. 2026 Jul 22;16(7):e115537. doi: 10.1136/bmjopen-2025-115537 (PMC13410707; doi:10.1136/bmjopen-2025-115537)
Supplement: Supplementary data [file bmjopen-16-7-s002.pdf]

Interview Guide: Asking About Violence in Child Health Services

Thank you for agreeing to participate in this interview. The aim of the study is to gain a deeper understanding of nurses' experiences of working with the identification of intimate partner violence within child health services. The focus is particularly on the experience of asking parents about violence.

Your responses will be treated confidentially and anonymized in the analysis and reporting. There are no right or wrong answers, I am interested in your personal experiences and reflections. The interview is expected to take approximately 30–60 minutes. You may withdraw from the interview at any time or skip any questions if you wish.

1. Characteristics of the intervention (the practice of asking about violence)

- How do you experience asking parents about violence within child health services?
- How clear do you find the guidelines regarding asking about violence?
- Do you think this way of working fits within your role as a child health nurse?
- What are your thoughts on asking both parents about violence?

2. Outer setting (external influences and support)

- How do parents usually respond when you ask about violence?
- Is there accessible support available for families who need it?
- How do you perceive collaboration with social services when making a report of concern?

3. Inner setting (organizational conditions)

- What kind of support is provided by management for working with issues related to violence at your workplace?
- Is there sufficient time and space during your meetings with parents to address questions about violence?
- What support is available at your workplace when making a report of concern in cases of suspected violence?

4. Characteristics of the individual (your own role and experience)

- How comfortable do you feel asking questions about violence?
- Have you received (sufficient) training, supervision, or other support to be able to talk about violence?

- Do you experience any differences in how you approach mothers compared to fathers when asking about violence?
- How do you feel about making a report of concern when you suspect that a child is being harmed?
- What do you think would make it easier for you to work with these issues?

5. Process (implementation of work with violence)

- How was the work of asking about violence introduced at your workplace?
- Are there opportunities within your team to discuss or reflect on these issues?
- How would you like the work with violence to develop in the future?

Closing

- Is there anything else you would like to add that we have not discussed?
- Is there anything you consider particularly important to highlight in this context?
