# Supplementary material for: Response to family violence in child health services (FRIDa): study protocol of a mixed-method study in the Stockholm region, Sweden
Source: BMJ Open. 2026 Jul 22;16(7):e115537. doi: 10.1136/bmjopen-2025-115537 (PMC13410707; doi:10.1136/bmjopen-2025-115537)
Supplement: Supplementary data [file bmjopen-16-7-s003.pdf]

Table 1. Timeline of the study

	2025		2026				2027				2028	
**Timepoint**	**Q3**	**Q4**	**Q1**	**Q2**	**Q3**	**Q4**	**Q1**	**Q2**	**Q3**	**Q4**	**Q1**	**Q2**
**Quantitative part: Register data and CHS clinic organizational data**												
Ethical approval	X											
Data collection		X	X									
Data management & analysis			X	X	X	X						
**Qualitative part: interview data**												
Ethical approval	X											
Recruitment							X					
Interviews							X	X				
Data management & analysis								X	X	X		
**Integration of the results of the quantitative and qualitative parts**												
Compilation & presentation of results											X	X
