# Supplementary material for: Response to family violence in child health services (FRIDa): study protocol of a mixed-method study in the Stockholm region, Sweden
Source: BMJ Open. 2026 Jul 22;16(7):e115537. doi: 10.1136/bmjopen-2025-115537 (PMC13410707; doi:10.1136/bmjopen-2025-115537)
Supplement: Reviewer comments [file bmjopen-2025-115537.reviewer_comments.pdf]

PEER REVIEW HISTORY

BMJ Open publishes all reviews undertaken for accepted manuscripts. Reviewers are asked to complete a checklist review form and are provided with free text boxes to elaborate on their assessment. These free text comments are reproduced below.

ARTICLE DETAILS

Title (Provisional)

Response to family violence in child health services (FRIDa): study protocol of a mixed method study in the Stockholm region, Sweden

Authors

Fröjlinger, Anna; Blom, Lisa; Tiitinen Mekhail, Kirsi; Lalouni, Maria; Lindberg, Lene

---

VERSION 1 - REVIEW

**Reviewer**	**1**
**Name**	**Garstang, Joanna**
**Affiliation**	**University of Birmingham**
**Date**	**23-Feb-2026**
**COI**	**None**

This is a well written protocol. I have no particular edits to suggest. I look forward to seeing the results of the study

---

**Reviewer**	**2**
**Name**	**Kalokhe, Ameeta**
**Affiliation**	**Emory University**
**Date**	**24-Mar-2026**
**COI**	**None**

The manuscript "Response to family violence in child health services (FRIDa): study protocol of a mixed method study in the Stockholm region, Sweden" describes a protocol for a mixed-methods study that 1) examines current implementation of family violence assessments among mothers and fathers in the CHS program in Sweden, 2) explores the CHS organizational framework for family violence assessment, and 3) qualitatively examines the experiences of nurses in assessing family violence. While the study has potential to address a question of high public health significance, the paper has several limitations.

Major Comments

• Strengths and Limitations: This statement needs to be clarified/reworded for readability: “Nurse selection is based on clinic-level variation to minimize perceived exposure, which may introduce bias if nurses with more positive attitudes toward routine questioning are more likely to participate.”

• Introduction: The introduction needs to be better organized. For example, authors should clearly delineate ‘witnessing’ FV verses ‘direct experience’ of FV up front and thereafter be consistent in use of these terms. The introduction also seems to go back-and-forth between screening/assessing for FV and interventions (i.e. referral) but really should focus on screening/assessment and associated gaps (as this seems to be most related to the objective of the study). Also, what is known about childhood exposure/witness of FV in Sweden? What is known about direct childhood FV experience? Are there published studies examining how FV is assessed in Sweden or worldwide – how does the present study contribute to existing literature?

• Methods: This statement needs clarification “However, data will only be retrieved from the records where 1) a parent has had an individual conversation, 2) where questions about violence have been raised or 3) where a report of concern has been made during the study period.” Why (if the aim is to assess frequency of participants in whom FV are asked) would you exclude the others?

• Analysis: this needs elaboration: “The statistical program SPSS will be used for statistical analyses to examine differences in asked questions and identify FV between mothers and fathers.” What analyses will be used? What is intended by “differences in asked questions”?

• The section “Quantitative workstream related to CHS organizational preconditions” needs more introduction. It seems arbitrarily added in the middle of the Methods. The analysis section also needs elaboration on what are the planned statistical analyses.

• The qualitative protocol: will interviews be recorded? Coded, and if so, will they be dually coded? How will coding disagreements be handled?

Minor Comments

• Abstract: In the statement, “Furthermore, a qualitative interview study will gain further information about the work with FV within the CHS,” “further information” should be more detailed.

• Introduction: “Recommendations by WHO are that services having regular contacts with women and children may ask questions about FV on routine while services with infrequent contacts could use questions about FV in presence of evidence-based risk factors” needs clarification

• Replace ‘prevalence’ with ‘frequency’

• Warrants English grammatical editing.

VERSION 1 - AUTHOR RESPONSE

Reviewer: 2
Dr. Ameeta Kalokhe, Emory University

Comments to the Author:
The manuscript “Response to family violence in child health services (FRIDa): study protocol of a mixed method study in the Stockholm region, Sweden” describes a protocol for a mixed-methods study that 1) examines current implementation of family violence assessments among mothers and fathers in the CHS program in Sweden, 2) explores the CHS organizational framework for family violence assessment, and 3) qualitatively examines the experiences of nurses in assessing family violence. While the study has potential to address a question of high public health significance, the paper has several limitations.

Major Comments

Comment 1: Strengths and Limitations: This statement needs to be clarified/reworded for readability: “Nurse selection is based on clinic-level variation to minimize perceived exposure, which may introduce bias if nurses with more positive attitudes toward routine questioning are more likely to participate.”

Response: Thank you for pointing this out. We have rephrased this sentence to improve clarity and readability, and to more clearly describe both the rationale for the sampling strategy and its potential limitations (Strengths and limitations, page 3, lines 56-59):

“Nurses will be purposively selected to reflect clinic-levels variation in routines for asking about FV. However, as recruitment occurs at the clinic level and participation is voluntary, there is at risk that nurses with more positive attitudes toward questioning about violence may be more likely to participate”

Comment 2: Introduction: The introduction needs to be better organized. For example, authors should clearly delineate ‘witnessing’ FV verses ‘direct experience’ of FV up front and thereafter be consistent in use of these terms. The introduction also seems to go back-and-forth between screening/assessing for FV and interventions (i.e. referral) but really should focus on screening/assessment and associated gaps (as this seems to be most related to the objective of the study). Also, what is known about childhood exposure/witness of FV in Sweden? What is known about direct childhood FV experience? Are there published studies examining how FV is assessed in Sweden or worldwide – how does the present study contribute to existing literature?

Response: Thank you for this constructive comment. We have restructured the Introduction to more clearly focus on FV identification and assessment within CHS, and have reduced emphasis on interventions, as these are not the primary focus of the present study. We have added a brief overview of existing Swedish research on childhood exposure to family violence, distinguishing between witnessing violence and direct victimization, and highlighted the limited knowledge regarding how such exposure is identified within CHS. We have also ensured consistent use of terminology throughout the manuscript and more clearly articulated how the present study contributes to existing literature (Please, see Introduction, page 4-7).

Comment 3: Methods: This statement needs clarification "However, data will only be retrieved from the records where 1) a parent has had an individual conversation, 2) where questions about violence have been raised or 3) where a report of concern has been made during the study period." Why (if the aim is to assess frequency of participants in whom FV are asked) would you exclude the others?

Response: Thank you for this important comment. We agree that this part requires clarification. All children registered at the included child health clinics during the study period constitute the study population, and information on the total number of registered children is available. This allows us to calculate the frequency of being asked about FV in relation to the full population. The restriction described refers only to which medical records are reviewed to extract detailed information. In the CHS context, documentation of questions about violence, individual parental consultations, or reports of concern are recorded in specific parts of the medical record. Therefore, data extraction is limited to records where such documentation is present to ensure that relevant information is captured accurately. Importantly, this does not imply that other children are excluded from the denominator when calculating frequencies. Rather, it reflects the structure of clinical documentation, where the absence of such entries indicates that no assessment or related documentation has been made.

We have clarified this in the Methods section (page 11, lines 256-262):

"Since questions about violence should be asked whenever there is a suspicion of violence, all medical records from the CHS of the two care providers are eligible for inclusion in the data collection. The total number of enrolled children will be used as the denominator when calculating frequencies. Information on FV assessment will be extracted from medical records where documentation of (1) an individual parental consultation, (2) questions about violence, or (3) a report of concern is present during the study period."

Comment 4: Analysis: this needs elaboration: "The statistical program SPSS will be used for statistical analyses to examine differences in asked questions and identify FV between mothers and fathers." What analyses will be used? What is intended by "differences in asked questions"?

Response: We have expanded and clarified the analysis section and integrated the two quantitative parts into a single analysis section. We now specify that descriptive statistics will be used to calculate the frequency of questions about family violence posed to parents, identification of ongoing violence, and reports of concern. Differences between mothers and fathers in being asked about violence will be examined using appropriate statistical tests. Furthermore, regression analyses will be conducted to explore associations between questions about violence and contextual variables such as Care Need Index (CNI) and, organizational and clinic-level factors (Methods, page 13, lines: 302-308)

"Descriptive statistics will be used to calculate the frequency of 1) questions about FV posed to parents, 2) identification of ongoing violence, and 3) reports of concern following identification. Differences between mothers and fathers in being asked about violence will be examined using appropriate statistical tests depending on the data distribution. Regression analyses will be conducted to explore associations between questions about violence and CNI, and organizational and clinic-level variables. All analyses will be conducted using the statistical software SPSS."

Comment 5: The section "Quantitative workstream related to CHS organizational preconditions" needs more introduction. It seems arbitrarily added in the middle of the Methods. The analysis section also needs elaboration on what are the planned statistical analyses.

Response: We appreciate these comments and acknowledge that both the structure of the quantitative part and the description of the planned statistical analyses needed to be clarified.

To address this, we have revised the structure of the Methods section by integrating the previously separate quantitative workstreams into a single, coherent quantitative part. Within this part, we now clearly describe the two data sources: (1) registry-based data from CHS medical records and (2) organizational data from CHS clinics part (Methods, pages 9-12).

We have also added a description of how these data sources will be combined at the clinic level (Methods, page 13, lines 297-299):

"Data from medical records on FV assessment will be linked to corresponding clinic-level variables, including CNI and organizational characteristics, to examine how contextual factors may be associated with assessment practices"

The statistical analysis section has also been expanded and clarified (see response to Comment 4), where we now specify the use of descriptive statistics and regression to examine associations between asking about violence and contextual factors.

Comment 6: The qualitative protocol: will interviews be recorded? Coded, and if so, will they be dually coded? How will coding disagreements be handled?

Response: Thank you for this important comment. Yes, all interviews will be audio-recorded and transcribed verbatim before analysis. The data will be analyzed using reflexive thematic analysis following Braun and Clarke (2022). In this approach, all researchers involved will read the transcripts and take part in the analytic process. Coding will be conducted in a flexible and iterative way, where researchers engage closely with the data and develop codes and themes through ongoing discussion. In line with reflexive thematic analysis, coding is not conducted independently for comparison, and differences in interpretation are not treated as discrepancies to be resolved by consensus. Instead, different interpretations are used to deepen the analysis through discussion and reflection.

We have expanded the description of the qualitative analysis in the Methods section, page 15, lines 350-357:

"The data will be analyzed using reflexive thematic analysis following Braun and Clarke (2022). The analysis will involve an iterative and recursive process, moving back and forth between reading the transcripts, coding the data, and developing themes. The researchers will read the transcripts and contribute to the analytic process through ongoing discussion. The focus of the analysis will be on exploring variations in nurses' experiences of asking about, identifying, and responding to FV within CHS"

We specify that interviews will be audio-recorded and transcribed verbatim on page 14, lines 339-340: "*The interviews will be audio-recorded and thereafter transcribed verbatim.*"

Minor Comments

Comment 7: Abstract: In the statement, "Furthermore, a qualitative interview study will gain further information about the work with FV within the CHS," "further information" should be more detailed.

Response: We have revised the sentence in the abstract to more clearly specify the aim of the qualitative component, clarifying that it will explore organizational conditions, as well as nurses' experiences of asking about, identifying, and responding to family violence within CHS (Abstract, page 2, lines 32-34):

"Furthermore, a qualitative interview study will gain information about the organizational barriers and facilitators, and nurses' professional experiences of working with FV within the CHS."

Comment 8: Introduction: "Recommendations by WHO are that services having regular contacts with women and children may ask questions about FV on routine while services with infrequent contacts could use questions about FV in presence of evidence-based risk factors" needs clarification

Response: Thank you for pointing this out. We have revised the wording of this statement to improve clarity and readability, and to more clearly reflect the WHO recommendations regarding routine versus targeted enquiry about family violence (Introduction, page 5, lines 101-104):

"WHO recommends that services with regular contact with women and children consider routine enquiry about FV, whereas services with less frequent contact may instead use targeted questions based on identified risk factors."

Comment 9: Replace 'prevalence' with 'frequency'

Response: We have replaced "prevalence" with "frequency" throughout the manuscript where appropriate, to better reflect the descriptive nature of the data.

Comment 10: Warrants English grammatical editing.

Response: The manuscript has been carefully revised for language and clarity, and minor grammatical errors have been corrected throughout.
