# Supplementary material for: Response to family violence in child health services (FRIDa): study protocol of a mixed-method study in the Stockholm region, Sweden
Source: BMJ Open. 2026 Jul 22;16(7):e115537. doi: 10.1136/bmjopen-2025-115537 (PMC13410707; doi:10.1136/bmjopen-2025-115537)
Supplement: Author's manuscript [file bmjopen-2025-115537.draft_revisions.pdf]

BMJ Open

BMJ Open is committed to open peer review. As part of this commitment we make the peer review history of every article we publish publicly available.

When an article is published we post the peer reviewers' comments and the authors' responses online. We also post the versions of the paper that were used during peer review. These are the versions that the peer review comments apply to.

The versions of the paper that follow are the versions that were submitted during the peer review process. They are not the versions of record or the final published versions. They should not be cited or distributed as the published version of this manuscript.

BMJ Open is an open access journal and the full, final, typeset and author-corrected version of record of the manuscript is available on our site with no access controls, subscription charges or pay-per-view fees (http://bmjopen.bmj.com).

If you have any questions on BMJ Open's open peer review process please email info.bmjopen@bmj.com

BMJ Open

Response to family violence in child health services (FRIDa): study protocol of a mixed method study in the Stockholm region, Sweden

Journal:	*BMJ Open*
Manuscript ID	bmjopen-2025-115537
Article Type:	Protocol
Date Submitted by the Author:	17-Dec-2025

Complete List of Authors:	Fröjlinger, Anna; Karolinska Institutet, Department of Global Public Health<br>Blom, Lisa; Karolinska Institute, Department of Global Public Health<br>Lalouni, Maria; Karolinska Institute, Dept of Clinical Neuroscience<br>Tiitinen Mekhail, Kirsi; Karolinska Institutet, Department of Neurobiology, Care Sciences and Society<br>Lindberg, Lene; Karolinska Institutet, Department of Global Public Health

Keywords:	Gender-Based Violence, Community child health < PAEDIATRICS, Child protection < PAEDIATRICS, Nursing research

SCHOLARONE™
MANUSCRIPTS

Response to family violence in child health services (FRIDa): study protocol of a mixed method study in the Stockholm region, Sweden

Anna Fröjlinger (0009-0006-5411-6633)[1], Lisa Blom (0000-0002-3538-7159)[1], Kirsi Tiitinen Mekhail (0000-0002-5715-8656)[2], Maria Lalouni (0000-0002-6818-8156)[3], Lene Lindberg (0000-0002-9275-551X)[1,4]

[1]Department of Global Public Health, Karolinska Institutet, Solnavägen 1E, 113 65 Stockholm, Sweden

[2] Department of Neurobiology, Care Sciences and Society, Alfred Nobels allé 23, 141 52 Huddinge, Sweden

[3] Department of Clinical Neuroscience, Karolinska Institutet, Tomtebodavägen 18A, 171 77 Stockholm, Sweden

[4] Center for Epidemiology and Community Medicine, Region Stockholm, Solnavägen 1E, 113 65 Stockholm, Sweden

Correspondance to: A Fröjlinger anna.frojlinger@ki.se

For peer review only - http://bmjopen.bmj.com/site/about/guidelines.xhtml

Abstract

- **Background**: Growing up with family violence (FV) is potentially harmful both in a short- and long-term perspective for the child. Early identification and support are therefore important. The purpose of this project (FRIDa) is to gain a deeper knowledge about identification of and response to FV in the Swedish Child Health Services (CHS). Further, we would like to better understand how structure and organization together with nurses' perceptions may contribute to identification and response within the CHS.

  Methods and analysis: In this part of the FRIDa-project, we will use a mixed method design to explore the pattern of identification of, and response to, FV. Approximately 90 000 CHS medical records over 12 months will be analyzed with non-linear regression to examine how FV identification and response vary by demographic factors. Furthermore, a qualitative interview study will gain further information about the work with FV within the CHS. The quantitative and qualitative results will be integrated in an explorative data analysis focusing on variations in the prevalence of posed questions, identification and response to FV supported by interview data.
- **Ethics and dissemination**: The study was approved by the Swedish Ethical Review Authority (Dnr 2025-03516-01). Results will be published with open access in international peer-review journals and communicated to national and regional CHS to contribute to the understanding of the long-term clinical work with FV within CHS. It will also add to the advancement of evidence-based practices for identification of, and response to, FV.

- **Trial registration**: ClinicalTrials.gov NCT07158983. Registered on August 28, 2025.

Strengths and limitations of this study

For peer review only

- This mixed-methods design combines large-scale register data with qualitative interviews, enabling both breadth and depth in understanding identification and response to FV within CHS.
- The inclusion of approximately 90,000 child health records provides strong statistical power for detecting patterns, although rare outcomes remain challenging to compare.
- Two major CHS care providers are included, covering clinics in areas with diverse socioeconomic conditions. However, this limits generalizability beyond the region.
- Nurse selection is based on clinic-level variation to minimize perceived exposure, which may introduce bias if nurses with more positive attitudes toward routine questioning are more likely to participate.

Keywords

Family Violence, Child Health Services, Identification, Support, Mixed Methods, Parental Support

For peer review only

For peer review only - http://bmjopen.bmj.com/site/about/guidelines.xhtml

Introduction

Family violence (FV) is a serious global public health concern [1]. The prevalence of FV varies depending on definition, how, when, and where data has been collected [2, 3]. Children can experience FV directly through physical, psychological or sexual abuse or indirectly when someone else in the family is the target of violence. Short- and long-term consequences for children experiencing FV have been described concerning physical health, cognitive, emotional and behavioral problems or disorders [4]. Another identified consequence associated with exposure to FV during childhood is the risk of being a victim or perpetrator of FV in adulthood [5].

Early detection and response to FV could prevent negative effects for children. Child health services (CHS) are one of several important services in this work in the Swedish context. Whether detection of FV should be administered through questions on routine or by indication is not evident. Research so far has failed to demonstrate that routine questions about FV reduce the prevalence of FV [6]. That may, however, be explained by lack of effective interventions, rather than by the identification process. Recommendations by WHO are that services having regular contacts with women and children may ask questions about FV on routine while services with infrequent contacts could use questions about FV in presence of evidence-based risk factors [7].

Experiencing violence against a close relative or loved one can be equally harmful to a child as being directly exposed to the violence, and the consequences are similar irrespective if it is the mother or father that is the victim or the perpetrator [8, 9]. Few studies have examined how questions on exposure to violence posed to new fathers in the healthcare setting are working. A few Swedish studies from the CHS context report a lower

For peer review only - http://bmjopen.bmj.com/site/about/guidelines.xhtml

share of fathers than mothers being asked about violence [10, 11]. To our knowledge, there are no international studies describing an approach such as the one in the study described herein where questions about their own exposure to violence are asked to new fathers from a violence prevention and parenting support perspective within healthcare. Furthermore, FV exists in all societies and cultures but there is an increased risk in areas with a higher degree of social vulnerability [12]. Examining whether there are differences in the extent to which parents in areas with different socioeconomic status are given the opportunity to answer questions about violence is therefore of relevance. Social vulnerability at a societal level can be measured in several ways. One such measure is Care Need Index (CNI), a socioeconomic index used to estimate healthcare needs within an area [13] It is based on factors such as single parenthood, unemployment, low educational level, and proportion of foreign-born residents. A higher CNI indicates a greater need for support and resources in primary health care.

Identification of FV does not always lead to actions being taken or decreased exposure to violence according to a Cochrane review [6]. A prerequisite for healthcare professionals to ask about exposure to violence is to feel confident about actions and steps to follow if violence is identified [14]. Structural and organizational preconditions such as training or supervision are also identified as factors affecting to which extent healthcare personnel are asking about exposure to violence [15]. Previous research on asking about FV in Swedish CHS has focused on the implementation phase [10, 11, 14]. In contrast, the present study examines the experiences of CHS nurses who have worked with this approach for several years, providing insights into long-term practice rather than initial adoption. Furthermore, the approach in this study includes asking both mothers and fathers in the same family about their own exposure to violence, which is uncommon in

For peer review only - http://bmjopen.bmj.com/site/about/guidelines.xhtml

previous research. This perspective is important for understanding how routine questions can support violence prevention and parenting support in a more inclusive way.

To sum up, exposure to FV among children is a severe public health problem that could be reduced by early identification and response in CHS. More research is needed to examine the opportunities of mothers and fathers and parents in different socioeconomic areas to answer questions on violence in CHS. There is also a need to increase the knowledge on the support needed among CHS nurses to ask about exposure to violence and respond when violence is identified, particularly from a long-term perspective after the approach has been established in practice.

Methods and analysis

Aim

The aim of this project is to gain a deeper knowledge on how FV is identified and responses to FV within the Swedish CHS. Further, we aim to understand how structure and organization together with nurses' perceptions may contribute to identification of, and response to FV within the CHS.

Research questions

The specific research questions are:

- To what extent are questions about FV posed to mothers and fathers, respectively, in CHS?
- To what extent is ongoing FV identified among parents that have been asked about it?

For peer review only - http://bmjopen.bmj.com/site/about/guidelines.xhtml

- To what extent have reports of concern been made to social services when FV is identified?
- To what extent are structural factors (e.g., CNI) and organizational conditions (e.g. professional education and supervision) associated with nurses' likelihood of inquiring about FV and to respond when FV is identified within CHS?"
- What barriers and facilitators do nurses within CHS perceive when asking about FV?
- What barriers and facilitators do nurses within CHS perceive in providing support to families affected by identified FV?

Design

The study will use a mixed method design including both quantitative and qualitative approaches (workstreams). In the quantitative workstreams of the study, data from CHS medical records will be used to identify the prevalence of questions posed on routine and based on indication and reports of FV within the CHS. Data from clinical managers are used to map the preconditions of the CHS clinics. The qualitative workstream of the study consists of interviews with nurses to explore their experiences of identifying and responding to identified FV. The combination of methods will support the understanding of the situation around working with identifying FV within CHS. The study protocol was developed according to the Good reporting of a mixed methods study (GRAMMS). GRAMMS is used as a checklist to ensure that relevant information is included in the study protocol and it will also be used in the reporting of the results [16].

Study setting

The study is set in the Stockholm region in Sweden and involve two of the caregivers

For peer review only - http://bmjopen.bmj.com/site/about/guidelines.xhtml

providing CHS in Stockholm region - Stockholm Health Care Services and Capio. Together they account for about half of the children at CHS clinics in the region. Stockholm comprises about 20% of all children born in Sweden. Sweden has a well-established national CHS program offered to all children 0-6 years of age free of charge. In addition to promoting health and development and preventing illness, the CHS program also aims to detect potential problems and intervene early to lay ground for healthy development. FRIDa is a research project aimed at supporting CHS in identifying and responding to FV through structured routines and staff training [17]. Based on the FRIDa model, routine questions about family violence have been implemented in the CHS in the Stockholm region. For the mother, this usually takes place during an individual visit when the child is 6-8 weeks and for the father at an individual visit when the child is 3-5 months. Three questions are asked, relating to 1) prior exposure to violence for the parent, 2) ongoing exposure to violence for the parent, and 3) ongoing exposure to violence for the child. Questions on exposure to violence should also be asked at any time based on indication, whenever there is a suspicion of violence.

Quantitative workstream related to CHS registries: prevalence of questions on FV posed to parents within CHS

Objectives

The objective of the quantitative workstream is to map questions about identification of FV in CHS in the Stockholm region by reviewing CHS medical records. A further objective is to gain knowledge on how nurses respond when FV is identified.

Participants

For the quantitative workstream we will make use of registry data from parents to children

For peer review only - http://bmjopen.bmj.com/site/about/guidelines.xhtml

enrolled in the CHS of two health care providers in the Stockholm region, namely Stockholm Health Care Services and Capio during the period 2024-06-30 to 2025-06-30. We estimate that the registry review will include about 90 000 records. The included CHS are based in areas with different socioeconomic characteristics (according to CNI) located in the city, suburbs and surrounding municipalities in the Stockholm region.

Measurements and data collection

De-identified patient journals from CHS will be compiled with support from administrators from the two care providers. There are specific variable names in the medical records for questions about exposure to violence, reports of concern to social services, and whether or not the mother or father had an individual visit with the CHS nurse.

The data that will be retrieved from the CHS medical records are: date for the visit; age of the child; type of contact; information about whether an individual visit has taken place for the parent (yes/no); whether the parent has been offered a conversation based on the Edinburgh Postnatal Depression Scale (EPDS) (yes/no); whether a question on exposure to violence has been asked (yes/no); notes about exposure to violence for the child (yes/no); reports of concern to social services (yes/no); and reason for reports of concern to social services (when a report is made).

Since questions on violence should be asked whenever there is a suspicion of violence, all medical records from the CHS of the two care providers will be included in the data collection. However, data will only be retrieved from the records where 1) a parent has had an individual conversation, 2) where questions about violence have been raised or 3) where a report of concern has been made during the study period.

For peer review only - http://bmjopen.bmj.com/site/about/guidelines.xhtml

Analysis

In this workstream, data from the records will be analyzed descriptively regarding questions asked about violence to parents, identification of ongoing violence and reports of concern made within a month from identification of FV. The statistical program SPSS will be used for statistical analyses to examine differences in asked questions and identify FV between mothers and fathers.

Quantitative workstream related to CHS organizational preconditions: background data of the included CHS clinics

Objectives

The objective of this part of the quantitative workstream is to map the preconditions of the included CHS clinics.

Sampling and recruitment

All unit managers and operations managers at the participating CHS clinics will be contacted.

Data collection

An electronic questionnaire with questions about organizational characteristics of the included CHS clinics will be distributed via a secure web platform. The questions will cover information on the number of nurses employed, their percentage of full-time employment, years of experience in CHS, number of enrolled children and how many of the nurses that have participated in training about FV. The questionnaire also includes questions on staff turnover in the latest year. It is estimated to take about 15 minutes to complete the

For peer review only - http://bmjopen.bmj.com/site/about/guidelines.xhtml

questionnaire. The CNI for each CHS clinic is publicly available and will be retrieved from Region Stockholm.

Informed consent

An information letter will be distributed to all unit managers and operations managers of the included CHS clinics. The information letter includes information about the voluntariness to complete the questionnaire, that all information will be reported on a group level without possibilities to identify specific CHS clinic or nurse and that the data collected will only be available to the researchers involved and stored securely at Karolinska Institutet.

Analysis

The data collected will be used to compare how the prevalence of asking questions and identifying ongoing FV correlates with CNI on an area level. The data will also be used to examine the correlation between organizational contextual factors and the work around identifying and responding in case of FV.

The qualitative workstream: nurses experiences around asking questions about, and responses, in case of FV in CHS

Objectives

The objective of the qualitative workstream is to gain insight into nurses' perspectives on, and experiences from, working with questions about violence in CHS and how it is embedded and sustained in the routine practice. An additional objective is to better understand how they respond when FV is identified.

For peer review only

Sampling and recruitment

Based on the results of the quantitative workstream, a selection of 10-15 nurses will be approached and asked for participation in semi-structured individual interviews. The selection will consist of nurses working at CHS clinics with different CNI and with high and low levels of parents having been asked questions about FV according to the results from the quantitative workstreams.

Data collection

Semi-structured individual interviews will be conducted focusing on barriers and facilitating factors to pose questions on exposure to violence and to respond when violence exposure is identified. The interview guide is informed by the Consolidated Framework for Implementation Research (CFIR) that can be used to support the understanding of factors affecting the implementation of evidence-based methods and interventions (Damschroder et al 2022). The interviews will be conducted face-to-face or digitally via Zoom or Teams after informed consent has been signed. The interviews will be audio recorded and thereafter transcribed verbatim.

Informed consent

Written informed consent will be obtained from the nurses prior to participating in the interviews. The nurses will be informed that participation is voluntary and that data will be presented in a way minimizing the risk of identifying individual participants. Nurses participating in the interviews will also have access to support from a psychologist from the study team.

Analysis

For peer review only - http://bmjopen.bmj.com/site/about/guidelines.xhtml

The interviews will be analyzed with thematic analysis. The focus of the analysis of the interview material will be on variations in the prevalence of posed questions, identification of and response to violence of FV.

Integration of the results from the quantitative and the qualitative workstreams

Results from the three workstreams will be integrated in an explorative data analysis with a mixed method approach (Fetters et al., 2013). The focus on the analysis will be to explore variations in the prevalence of posed questions, identification and response to FV supported by interview data.

Study timeline

The timeline of the study is described in Table 1. Ethical approval was sought and granted in May 2025. The care givers will be approached and requested for patient data during October/November 2025. Data management and analysis of the register data will be initiated in November 2025 and will be finalized in November 2026. Nurses at selected CHS clinics will be contacted with written information and invitation to participate in interviews in January 2027. Interviews are planned to be conducted in Mars-June 2027. Data management and analysis of the interview data will be conducted in Mars-December 2027. The integration of the results of the material from the registry review and the interviews will be compiled and presented in January-June 2028.

Table 1. Timeline of the study

	2025		2026				2027				2028	
**Timepoint**	**Q3**	**Q4**	**Q1**	**Q2**	**Q3**	**Q4**	**Q1**	**Q2**	**Q3**	**Q4**	**Q1**	**Q2**
**Quantitative workstreams: Register data and characteristics of the CHS clinics**												
Ethical approval	X											

For peer review only

Data collection		X										
Data management & analysis		X	X	X	X	X						
**Qualitative workstream: interview data**												
Ethical approval	X											
Recruitment							X					
Interviews							X	X				
Data management & analysis								X	X	X		
**Integration of the results of the quantitative and qualitative workstreams**												
Compilation & presentation of results											X	X

Ethics and dissemination

The study was approved by the Swedish Ethical Review Authority (Dnr 2025-03516-01). Nurses will provide written informed consent before participating in the interviews. All results will be presented at a group level. The final data set will be handled in accordance with the guidelines of Karolinska Institutet. The variable list, the interview guide, the questionnaire to the clinical managers and the information letters are available from the corresponding author on request.

The results of the study will be published with open access in peer-reviewed journals and presented to regional and national CHS.

Patient and public involvement

Patients and the public were not involved in the design, conduct, reporting, or dissemination plans of this research. However, the results will be shared with child health service staff to inform practice.

For peer review only

Discussion

This protocol describes a mixed methods study designed to gain a deeper knowledge on identification of, and response to, FV in the Swedish CHS. Considering the serious health consequences that violence can cause, it is important that healthcare introduces evidence-based methods that facilitate identification and provide opportunities for support to victims of violence.

There are several practical and operational issues to discuss and reflect on in the upcoming study. One relates to the two outcomes “notes about exposure to violence for the child” and “reports of concern to social services”. Both outcomes are rare events which can make comparisons difficult, as reported in a previous study within the research group [17]. The large material in this study is expected to cater for better comparisons.

Another issue concerns the selection of CHS clinics. Only two care providers of CHS are included due to practical and logistical reasons. However, the two care providers together account for a large share of the CHS clinics in the uptake area and include clinics located in areas with varying socioeconomic conditions and are expected to provide a representative sample of the CHS clinics in the region.

Several considerations related to the selection of nurses and the interviews should also be lifted. The selection of nurses is made with the purpose of providing information from a variation of CHS clinics with different CNI and with low and high levels of parents that have been asked about exposure to violence. The nurses are selected based on the level of the CHS clinic instead of the individual nurses’ levels, in order to decrease the risk of nurses

For peer review only - http://bmjopen.bmj.com/site/about/guidelines.xhtml

feeling exposed. A downside of that is that we will not be able to control the selection of the individual nurses where nurses with a more positive perspective on asking questions around violence might be more willing to participate in the interviews. To mitigate this, the study emphasizes that interviews focus on barriers and facilitators and that confidentiality will be strictly maintained. Another concern is the choice of interviewer which may affect how comfortable nurses feel about sharing their experiences and perspectives. We will strive to select an interviewer that has no, or limited, connection to the day-to-day work of the nurses.

The study is expected to support the possibilities for children's and parents' equal opportunities for support via the CHS in case of FV. This is expected to be achieved by increasing knowledge about potential differences in how questions on violence are posed to parents related to gender, area level socioeconomic background and different possibilities, both actual preconditions and experienced opportunities, for nurses to comply with the method implemented. A strength of the study is its focus on nurses who have worked with the method for several years, offering insights into sustainability and normalization of the approach beyond the initial implementation phase. This long-term perspective is important for understanding not only feasibility but also how the method becomes embedded in routine practice. The findings may inform strategies for organizational support and training, ensuring that CHS nurses have the necessary conditions to maintain this practice over time. This could strengthen the capacity of CHS to identify FV early and provide equal opportunities for support to all parents, contributing to violence prevention and safer environments for children.

Trial status

For peer review only - http://bmjopen.bmj.com/site/about/guidelines.xhtml

Study protocol version number 2, December 15, 2025. Recruitment started in December 2025 and is estimated to be completed in March 2027.

Abbreviations

CHS – Child health services

CNI – Care need index

EPDS – Edinburgh Postnatal Depression Scale

FV – Family violence

Authors' contributions

AF and LB drafted the manuscript. LL is the principal investigator, and she conceived the study together with AF. All authors (AF, LB, KTM, ML and LL) have contributed to the study design and have critically revised the draft, read and approved the final manuscript.

Funding

This work was supported by the Swedish Research Council for Health, Working life and Welfare (Forte) Dnr: 2023-01144. Open access funding was provided by Karolinska Institutet. The funders had no role in the design of the study and will have no role in the data collection, analysis and interpretation or in writing the manuscript.

Competing interests

The authors declare that they have no competing interests.

References

For peer review only

For peer review only - http://bmjopen.bmj.com/site/about/guidelines.xhtml

1. Krug E, Dahlberg L, Mercy J, Zwi A, Lozano R. World report on violence and health. Geneva: World Health Organization; 2002.

2. UNICEF. A Familiar Face: Violence in the lives of children and adolescents.; 2017.

3. World Health Organization. Violence against women prevalence estimates, 2018: global, regional and national prevalence estimates for intimate partner violence against women and global and regional prevalence estimates for non-partner sexual violence against women. Geneva; 2021.

4. Howell KH, Barnes SE, Miller LE, Graham-Bermann SA. Developmental variations in the impact of intimate partner violence exposure during childhood. J Inj Violence Res. 2016;8(1):43-57. doi:10.5249/jivr.v8i1.663

5. Ehrensaft MK, Langhinrichsen-Rohling J. Intergenerational transmission of intimate partner violence: Summary and current research on processes of transmission. In: Geffner R, White JW, Hamberger LK, Rosenbaum A, Vaughan-Eden V, Vieth VI, editors. Handbook of interpersonal violence and abuse across the lifespan: Springer; 2022.

6. O'Doherty L, Hegarty K, Ramsay J, Davidson LL, Feder G, Taft A, et al. Screening women for intimate partner violence in healthcare settings. Cochrane database of systematic reviews. 2015;2015(8):CD007007. doi:10.1002/14651858.CD007007.pub3

7. World Health Organization. Responding to intimate partner violence and sexual violence against women: WHO clinical and policy guidelines. 2013.

8. Holt S, Buckley H, Whelan S. The impact of exposure to domestic violence on children and young people: A review of the literature. Child abuse & neglect. 2008;32(8):797-810. doi:10.1016/j.chiabu.2008.02.004

For peer review only - http://bmjopen.bmj.com/site/about/guidelines.xhtml

9. McTavish JR, MacGregor JCD, Wathen CN, MacMillan HL. Children's exposure to intimate partner violence: an overview. International review of psychiatry (Abingdon, England). 2016;28(5):504-18. doi:10.1080/09540261.2016.1205001

10. Nimborg J, Lindskog U, Nordgren L, Annerbäck EM. Routine conversations about violence conducted in Swedish child health services—A mixed methods study of nurses' experiences. Acta Paediatrica. 2023;112(3):442-51. doi:10.1111/apa.16355

11. Engström M, Lindqvist S, Janson S, Feldman I, Dubowitz H, Lucas S. Validation of the Swedish version of the safe environment for every kid (SEEK) parent screening questionnaire. BMC public health. 2023;23(1):1-11. doi:10.1186/s12889-023-16792-4

12. Capaldi DM, Knoble NB, Shortt JW, Kim HK. A Systematic Review of Risk Factors for Intimate Partner Violence. Partner abuse. 2012;3(2):231-80. doi:10.1891/1946-6560.3.2.231

13. Sundquist K, Malmström M, Johansson S, Sundquist J. Care Need Index, a useful tool for the distribution of primary health care resources. J Epidemiol Community Health. 2003;57(5):347-52. doi:10.1136/jech.57.5.347

14. Anderzen-Carlsson A, Gillå C, Lind M, Almqvist K, Lindgren Fändriks A, Källström Å. Child healthcare nurses' experiences of asking new mothers about intimate partner violence. Journal of clinical nursing. 2018;27(13-14):2752-62. doi:10.1111/jocn.14242

15. García-Moreno C, Hegarty K, d'Oliveira AF, Koziol-McLain J, Colombini M, Feder G. The health-systems response to violence against women. Lancet. 2015;385(9977):1567-79. doi:10.1016/s0140-6736(14)61837-7

16. O'Cathain A, Murphy E, Nicholl J. The quality of mixed methods studies in health services research. Journal of health services research & policy. 2008;13(2):92-8. doi:10.1258/jhsrp.2007.007074

For peer review only

For peer review only - http://bmjopen.bmj.com/site/about/guidelines.xhtml

17. Fröjlinger A, Lalouni M, Blom L, Tiitinen Mekhail K, Lindberg L. Identification and response to family violence within Swedish Child Health Services: a pragmatic randomized controlled trial. [manuscript submitted for publication]. 2025.

For peer review only

BMJ Open

Response to family violence in child health services (FRIDa): study protocol of a mixed method study in the Stockholm region, Sweden

Journal:	*BMJ Open*
Manuscript ID	bmjopen-2025-115537.R1
Article Type:	Protocol
Date Submitted by the Author:	25-May-2026
Complete List of Authors:	Fröjlinger, Anna; Karolinska Institutet, Department of Global Public Health<br>Blom, Lisa; Karolinska Institute, Department of Global Public Health<br>Tiitinen Mekhail, Kirsi; Department of Neurobiology, Care Sciences and Society<br>Lalouni, Maria; Karolinska Institute, Dept of Clinical Neuroscience; Region Stockholm, Center for Epidemiology and Community Medicine<br>Lindberg, Lene; Karolinska Institutet, Department of Global Public Health; Region Stockholm, Center for Epidemiology and Community Medicine
**Primary Subject Heading**:	Health services research
Secondary Subject Heading:	Public health, Paediatrics
Keywords:	Gender-Based Violence, Child protection < PAEDIATRICS, Nursing research, Community child health < PAEDIATRICS

SCHOLARONE™
MANUSCRIPTS

Response to family violence in child health services (FRIDa): study protocol of a mixed method study in the Stockholm region, Sweden

Anna Fröjlinger (0009-0006-5411-6633)[1], Lisa Blom (0000-0002-3538-7159)[1], Kirsi Tiitinen Mekhail (0000-0002-5715-8656)[2], Maria Lalouni (0000-0002-6818-8156)[3,4], Lene Lindberg (0000-0002-9275-551X)[1,4]

[1]Department of Global Public Health, Karolinska Institutet, Solnavägen 1E, 113 65 Stockholm, Sweden

[2] Department of Neurobiology, Care Sciences and Society, Alfred Nobels allé 23, 141 52 Huddinge, Sweden

[3] Department of Clinical Neuroscience, Karolinska Institutet, Tomtebodavägen 18A, 171 77 Stockholm, Sweden

[4] Center for Epidemiology and Community Medicine, Region Stockholm, Solnavägen 1E, 113 65 Stockholm, Sweden

Correspondence to: A Fröjlinger anna.frojlinger@ki.se

Word count (main text only): 3318

For peer review only

Abstract

Introduction: Growing up with family violence (FV) is potentially harmful both in a short- and long-term perspective for the child. Early identification and support are therefore important. The purpose of this project (FRIDa) is to gain a deeper knowledge about identification of and response to FV in the Swedish Child Health Services (CHS). Further, we would like to better understand how structure and organization together with nurses' perceptions may contribute to identification and response within the CHS.

Methods and analysis: In this part of the FRIDa-project, we will use a mixed method design to explore the pattern of identification of, and response to, FV. Approximately 90 000 CHS medical records over 12 months will be analyzed with regression analysis to examine how FV identification and response vary by demographic factors. Furthermore, a qualitative interview study will gain information about the organizational barriers and facilitators, and nurses' professional experiences of working with FV within the CHS. The quantitative and qualitative results will be integrated in an explorative data analysis focusing on variations in the frequencies of posed questions, identification and response to FV supported by interview data.

Ethics and dissemination: The study was approved by the Swedish Ethical Review Authority (Dnr 2025-03516-01). Results will be published with open access in international peer-review journals and communicated to national and regional CHS to contribute to the understanding of the long-term clinical work with FV within CHS. It will also add to the advancement of evidence-based practices for identification of, and response to, FV.

Trial registration: ClinicalTrials.gov NCT07158983. Registered on August 28, 2025.

Article summary

For peer review only

Strengths and Limitations

- This mixed-methods design combines large-scale register data with qualitative interviews, enabling both breadth and depth in understanding identification and response to FV within CHS.
- The inclusion of approximately 90 000 child health records provides strong statistical power for detecting patterns, although rare outcomes remain challenging to compare.
- Two major CHS care providers are included, covering clinics in areas with diverse socioeconomic conditions. However, this limits generalizability beyond the region.
- Nurses will be purposively selected to reflect clinic-level variation in routines for asking about FV. However, as recruitment occurs at the clinic level and participation is voluntary, there is a risk that nurses with more positive attitudes toward questioning about violence may be more likely to participate.

Keywords

Family Violence, Child Health Services, Identification, Support, Mixed Methods, Parental Support

For peer review only

Introduction

Family violence (FV) is a serious global public health concern [1]. The prevalence of FV varies depending on definition, how, when, and where data has been collected [2,3]. Children may experience FV through direct exposure (e.g. physical, psychological or sexual abuse), or by witnessing violence between caregivers or other family members. While these forms of exposure often co-occur, they represent conceptually distinct experiences [4]. Globally, a large proportion of children are exposed to FV [2]. In Sweden, self-reported data from adolescents indicate that 12 and 13% have been subjected to psychological or physical abuse by a caregiver, respectively, and around 10% have witnessed violence between adults in the home [5].

Short- and long-term consequences for children experiencing FV have been described concerning physical health, cognitive, emotional and behavioral problems or disorders [6]. Another identified consequence associated with exposure to FV during childhood is the risk of being a victim or perpetrator of FV in adulthood [7]. The consequences may be similar whether the child has witnessed violence between adults or has been directly exposed to violence [8].

Early identification of FV within child health services (CHS) is considered an important strategy for mitigating negative effects for children. In Sweden, CHS is a nurse-led, universal service offered free of charge to all children aged 0–5 years and their parents, reaching almost all families. All parents are offered an individual consultation with a CHS nurse during the child's first six months, providing an important opportunity to ask about exposure to FV. The high coverage and repeated contacts over time make CHS a central arena for identifying FV [9].

Whether detection of FV should be administered through questions on routine or by indication is not evident. Research so far has failed to demonstrate that routine questions

For peer review only

about FV reduce the prevalence of FV [10]. That may, however, be explained by lack of effective interventions, rather than by the identification process. WHO recommends that services with regular contact with women and children consider routine enquiry about FV, whereas services with less frequent contact may instead use targeted questions based on identified risk factors [11]

Few studies have examined how questions on exposure to violence posed to new fathers in the healthcare settings are working. A few Swedish studies from the CHS context report a lower share of fathers than mothers being asked about violence [12,13]. To our knowledge, no international studies have investigated how fathers are offered the opportunity to answer questions about their own exposure to violence within a child health or parenting support context.

FV exists in all societies and cultures but there is an increased risk in areas with a higher degree of social vulnerability [14]. Examining whether parents in areas with different socioeconomic conditions are equally likely to be asked about FV is therefore important. Social vulnerability at a societal level can be measured in several ways. One such measure used within CHS is Care Need Index (CNI), a socioeconomic index used to estimate healthcare needs within an area [15]. It is based on factors such as single parenthood, unemployment, low educational level, and proportion of foreign-born residents in the area. A higher CNI indicates a greater need for support and resources in primary health care.

Maintaining routines for asking about violence over time can be difficult, as these practices are often influenced by factors such as workload, staff turnover, and access to training. Even routines that are initially well implemented may diminish or become less consistently applied over time [16]. These organizational factors may also influence healthcare

For peer review only - http://bmjopen.bmj.com/site/about/guidelines.xhtml

professionals´ confidence in addressing violence, including their preparedness to act when violence is identified. Previous research suggests that a prerequisite for healthcare professionals to ask about exposure to violence is to feel confident about actions and steps to follow if violence is identified [17]. In Swedish CHS, existing research has focused on the implementation phase of such routines [12,13,17]. Less is known about how these practices are sustained over time and how nurses experience working with them in everyday clinical practice.

The present study addresses this gap by examining the experiences of CHS nurses who have worked with this approach for several years, providing insights into long-term practice rather than initial adoption. Furthermore, the approach in this study includes asking both mothers and fathers in the same family about their own exposure to violence, which is uncommon in previous research. This perspective is important for understanding how routine questions can support violence prevention and parenting support in a more inclusive way.

To sum up, exposure to FV among children is a severe public health problem that could be reduced by early identification and response in CHS. More research is needed to examine the opportunities of mothers and fathers, and parents in different socioeconomic areas to be asked about violence during CHS encounters. There is also a need for increased knowledge on the support CHS nurses require to ask about violence and respond when violence is identified, particularly from a long-term perspective.

Methods and analysis

Aim

For peer review only - http://bmjopen.bmj.com/site/about/guidelines.xhtml

The aim of this project is to gain a deeper knowledge on how FV is identified and responses to FV within the Swedish CHS. Further, we aim to understand how structure and organization together with nurses' perceptions may contribute to identification of and response to FV within the CHS.

Research questions

The specific research questions are:

- To what extent are questions about FV posed to mothers and fathers, respectively, in CHS?
- To what extent is ongoing FV identified among parents that have been asked about it?
- To what extent have reports of concern been made to social services when FV is identified?
- To what extent are structural factors (e.g., CNI) and organizational conditions (e.g. professional education and supervision) associated with nurses' likelihood of inquiring about FV and responding when FV is identified within CHS?"
- What barriers and facilitators do nurses within CHS perceive when asking about FV?
- What barriers and facilitators do nurses within CHS perceive in providing support to families affected by identified FV?

Design

The study will use a mixed method design including both quantitative and qualitative approaches. In the quantitative part of the study, data from CHS medical records will be used to identify the frequency of questions posed on routine and based on indication and

For peer review only - http://bmjopen.bmj.com/site/about/guidelines.xhtml

reports of FV within the CHS. Data from clinical managers are used to map the organizational preconditions of the CHS clinics. The qualitative part of the study consists of interviews with nurses to explore their experiences of identifying and responding to identified FV. The combination of methods will support the understanding of the situation around working with identifying FV within CHS. The study protocol was developed according to the Good Reporting of a Mixed Methods Study (GRAMMS). GRAMMS is used as a checklist to ensure that relevant information is included in the study protocol and it will also be used in the reporting of the results [18].

Study setting

The study is set in the Stockholm region in Sweden and involve two of the largest caregivers providing CHS in Stockholm region - Stockholm Health Care Services and Capio. Together they account for about half of the children enrolled at CHS clinics in the region. The Stockholm region comprises about 20% of all children residing in Sweden. FRIDa is a research project aimed at supporting CHS in identifying and responding to FV through structured routines and staff training [19]. Based on the FRIDa model, routine questions about FV have been implemented in the CHS in the Stockholm region. For the mother, this usually takes place during an individual visit when the child is 6-8 weeks and for the father at an individual visit when the child is 3-5 months. Three questions are asked, relating to 1) prior exposure to FV for the parent, 2) ongoing exposure to FV for the parent, and 3) ongoing exposure to FV for the child. Questions on exposure to violence should also be asked at any time based on indication, whenever there is a suspicion of violence.

Quantitative part

For peer review only - http://bmjopen.bmj.com/site/about/guidelines.xhtml

Objectives

The objective of the quantitative part of the study is to examine how questions about FV are asked within CHS in the Stockholm region, and how healthcare professionals respond when violence is identified. A further objective is to examine how these practices are associated with organizational and contextual factors at the CHS clinic level.

Data sources

The quantitative part will draw on two complementary data sources:

1. ***Registry-based data from CHS medical records***

Participants

We will make use of registry data from medical records of children enrolled in the CHS during the period 2024-06-30 to 2025-06-30. Approximately 90 000 children are enrolled in the CHS provided by the two included health care providers. The included CHS clinics are based in areas with different socioeconomic characteristics (according to CNI) located in the city, suburbs and surrounding municipalities in the Stockholm region.

Measurements and data collection

De-identified medical records from CHS will be compiled with support from administrators at the two care providers. There are specific variable names in the medical records for questions about exposure to violence, reports of concern to social services, and whether the mother or father had an individual visit with the CHS nurse.

The data that will be retrieved from the CHS medical records are: date for the visit; age of

For peer review only

the child; type of contact; information about whether an individual visit has taken place for the parent (yes/no); whether a question on exposure to violence has been asked (yes/no) and the parents' answer; notes about exposure to violence for the child (yes/no); reports of concern to social services (yes/no); and reason for reports of concern to social services (when a report is made).

Since questions about violence should be asked whenever there is a suspicion of violence, all medical records from the CHS of the two care providers are eligible for inclusion in the data collection. The total number of enrolled children will be used as the denominator when calculating frequencies. Information on FV assessment will be extracted from medical records where documentation of 1) an individual parental consultation, 2) questions about violence, or 3) a report of concern is present during the study period.

2. Organizational characteristics of CHS clinics

Sampling and recruitment

All unit managers and operations managers at the participating CHS clinics will be contacted.

Data collection

An electronic questionnaire with questions about organizational characteristics of the included CHS clinics will be distributed via a secure web platform (see supplementary file 1). The questions cover information on the number of nurses employed, their percentage of full-time employment, years of experience in CHS, number of enrolled children and how

For peer review only - http://bmjopen.bmj.com/site/about/guidelines.xhtml

many of the nurses that have participated in training about FV. The questionnaire also includes questions on staff turnover in the latest year. It is estimated to take about 15 minutes to complete the questionnaire. The CNI for each CHS clinic is publicly available and will be retrieved from Region Stockholm.

Informed consent

An information letter will be distributed to all unit managers and operations managers of the included CHS clinics. The information letter includes information about the voluntariness to complete the questionnaire, that all information will be reported on a group level without possibilities to identify specific CHS clinics or nurses and that the data collected will only be available to the researchers involved and stored securely at Karolinska Institutet.

Data integration

Data from medical records on FV assessment will be linked to corresponding clinic-level variables, including CNI and organizational characteristics, to examine how contextual factors may be associated with assessment practices.

Analysis

Descriptive statistics will be used to calculate the frequency of 1) questions about FV posed to parents, 2) identification of ongoing violence, and 3) reports of concern following identification. Differences between mothers and fathers in being asked about violence will be examined using appropriate statistical tests depending on the data distribution. Regression analyses will be conducted to explore associations between questions about

For peer review only

For peer review only - http://bmjopen.bmj.com/site/about/guidelines.xhtml

violence, CNI and organizational and clinic-level variables. All analyses will be conducted using the statistical software SPSS.

The qualitative part

Objectives

The objective of the qualitative part of the study is to gain insight into nurses' perspectives on, and experiences from, working with questions about FV in CHS and how it is embedded and sustained in the routine practice. An additional objective is to better understand how they respond when FV is identified.

Sampling and recruitment

Based on the results of the quantitative part, a selection of 10-15 nurses will be approached and asked for participation in semi-structured individual interviews. The selection will consist of nurses working at CHS clinics with different CNI and with high and low frequencies of parents being asked about FV according to the results from the quantitative part.

Data collection

Semi-structured individual interviews will be conducted focusing on barriers and facilitating factors to pose questions on exposure to violence and to respond when violence exposure is identified. The interview guide (see Supplementary file 2) is informed by the Consolidated Framework for Implementation Research (CFIR) that can be used to support the understanding of factors affecting the implementation of evidence-based methods and interventions [16]. The guide includes questions addressing experiences of asking about violence, perceived clarity of guidelines, organizational support and conditions,

For peer review only - http://bmjopen.bmj.com/site/about/guidelines.xhtml

collaboration with external actors, as well as individual confidence and preparedness to ask about and respond to violence. The interviews will be conducted face-to-face or digitally via Zoom or Teams after informed consent has been signed. The interviews will be audio-recorded and thereafter transcribed verbatim.

Informed consent

Written informed consent will be obtained from the nurses prior to participating in the interviews. The nurses will be informed that participation is voluntary and that data will be presented in a way minimizing the risk of identifying individual participants. Nurses participating in the interviews will also have access to support from a psychologist from the study team.

Analysis

The interviews will be analyzed using reflexive thematic analysis [20]. The analysis will involve an iterative and recursive process, moving back and forth between reading the transcripts, coding the data, and developing themes. The researchers will read the transcripts and contribute to the analytic process through ongoing discussion. The focus of the analysis will be on exploring variations in nurses' experiences of asking about, identifying, and responding to FV within CHS.

Integration of the results from the quantitative and the qualitative parts

Results from the two parts will be integrated in an explorative data analysis with a mixed method approach [21]. The focus on the analysis will be to explore variations in the frequency of questions posed, identification and response to FV supported by interview data.

For peer review only - http://bmjopen.bmj.com/site/about/guidelines.xhtml

Study timeline

The timeline of the study is described in Table 1. Ethical approval was sought and granted in May 2025. The care givers were approached and requested for patient- and organizational data during October/November 2025. Data management and analysis of the register data was initiated in February 2026 and will be finalized in November 2026. Nurses at selected CHS clinics will be contacted with written information and invitation to participate in interviews in January 2027. Interviews are planned to be conducted in March-June 2027. Data management and analysis of the interview data will be conducted in March-December 2027. The integration of the results of the material from the registry review and the interviews will be compiled and presented in January-June 2028.

Table 1. Timeline of the study

	**2025**		**2026**				**2027**				**2028**	
**Timepoint**	**Q3**	**Q4**	**Q1**	**Q2**	**Q3**	**Q4**	**Q1**	**Q2**	**Q3**	**Q4**	**Q1**	**Q2**
**Quantitative part: Register data and CHS clinic organizational data**												
Ethical approval	X											
Data collection		X	X									
Data management & analysis			X	X	X	X						
**Qualitative part: interview data**												
Ethical approval	X											
Recruitment							X					
Interviews							X	X				
Data management & analysis								X	X	X		
**Integration of the results of the quantitative and qualitative parts**												
Compilation & presentation of results											X	X

For peer review only

For peer review only - http://bmjopen.bmj.com/site/about/guidelines.xhtml

Ethics and dissemination

The study was approved by the Swedish Ethical Review Authority (Dnr 2025-03516-01). Nurses will provide written informed consent before participating in the interviews. All results will be presented at a group level. The final data set will be handled in accordance with the guidelines of Karolinska Institutet. The variable list and the information letters are available from the corresponding author on request.

The results of the study will be published with open access in peer-reviewed journals and presented to regional and national CHS.

Patient and public involvement

Patients and the public were not involved in the design, conduct, reporting, or dissemination plans of this research. However, the results will be shared with CHS staff to inform practice.

Discussion

This protocol describes a mixed methods study designed to gain a deeper knowledge on identification of, and response to, FV within the Swedish CHS. Considering the serious health consequences that violence can cause, it is important that healthcare introduces evidence-based methods that facilitate identification and ensure equitable opportunities for support for victims of violence.

There are several practical and operational issues to discuss and reflect on in the upcoming study. One relates to the two outcomes identification of exposure to violence for

For peer review only - http://bmjopen.bmj.com/site/about/guidelines.xhtml

and reports of concern to social services. Both outcomes are rare events which can make comparisons difficult, as reported in a previous study within the research group [19]. The large material in this study is expected to cater for better comparisons.

Another issue concerns the selection of CHS clinics. Only two care providers of CHS are included due to practical and logistical reasons. However, the two care providers together account for a large share of the CHS clinics in the uptake area and include clinics located in areas with varying socioeconomic conditions and are expected to provide a representative sample of the CHS clinics in the region.

Several considerations related to the selection of nurses and the interviews should also be lifted. The selection of nurses is made with the purpose of providing information from a variation of CHS clinics with different CNI and with low and high frequencies of parents that have been asked about exposure to violence. The nurses are selected based on the level of the CHS clinic instead of the individual nurses' levels, to decrease the risk of nurses feeling exposed. A downside of that is that we will not be able to control the selection of the individual nurses at a specific clinic, where nurses with a more positive perspective on asking questions around violence might be more willing to participate in the interviews. To mitigate this, the study emphasizes that interviews focus on barriers and facilitators and that confidentiality will be strictly maintained. Another concern is the choice of interviewer which may affect how comfortable nurses feel about sharing their experiences and perspectives. We will strive to select an interviewer that has no, or limited, connection to the day-to-day work of the nurses.

The study is expected to support the possibilities for children's and parents' equal

For peer review only

For peer review only - http://bmjopen.bmj.com/site/about/guidelines.xhtml

opportunities for support via the CHS in case of FV. This is expected to be achieved by increasing knowledge about potential differences in how questions on violence are posed to parents related to gender, area level socioeconomic background and different possibilities, both actual preconditions and experienced opportunities, for nurses to comply with the method implemented. A strength of the study is its focus on nurses who have worked with the method for several years, offering insights into sustainability and normalization of the approach beyond the initial implementation phase. This long-term perspective is important for understanding not only feasibility but also how the method becomes embedded in routine practice. The findings may inform strategies for organizational support and training, ensuring that CHS nurses have the necessary conditions to maintain this practice over time. This could strengthen the capacity of CHS to identify FV early and provide equal opportunities for support to all parents, contributing to violence prevention and safer environments for children.

Abbreviations

CHS – Child health services

CNI – Care need index

EPDS – Edinburgh Postnatal Depression Scale

FV – Family violence

Author contributions

AF and LB drafted the manuscript. LL is the principal investigator, and she conceived the study together with AF. All authors (AF, LB, KTM, ML and LL) have contributed to the

For peer review only - http://bmjopen.bmj.com/site/about/guidelines.xhtml

study design and have critically revised the draft, read and approved the final manuscript.

Funding

This work was supported by the Swedish Research Council for Health, Working life and Welfare (Forte) Dnr: 2023-01144. Open access funding was provided by Karolinska Institutet. The funders had no role in the design of the study and will have no role in the data collection, analysis and interpretation or in writing the manuscript.

Competing interests

The authors declare that they have no competing interests.

References

1. Krug EG, Mercy JA, Dahlberg LL, Zwi AB. The world report on violence and health. Geneva; 2002.

2. UNICEF. A Familiar Face: Violence in the lives of children and adolescents. New York; 2017.

3. World Health Organization. Violence against women prevalence estimates, 2018: global, regional and national prevalence estimates for intimate partner violence against women and global and regional prevalence estimates for non-partner sexual violence against women. Geneva: World Health Organization; 2021.

4. Howell KH, Taylor NR, Thomsen KN, Martinez-Torteya C, Källström Å. Global Perspectives on Family Violence: Prevalence and Effects on Children across Cultural Contexts. Psychological Perspectives on Understanding and Addressing Violence Against Children2022.

For peer review only

5. Jernbro C, Landberg Å, Thulin J. Våld mot barn 2022: En nationell kartläggning. Stockholm; 2023. Report No.: 91-86759-57-4978-91-86759-57-5.

6. Howell KH, Barnes SE, Miller LE, Graham-Bermann SA. Developmental variations in the impact of intimate partner violence exposure during childhood. Journal of injury and violence research. 2016;8(1):43-57.

7. Ehrensaft MK, Langhinrichsen-Rohling J. Intergenerational Transmission of Intimate Partner Violence: Summary and Current Research on Processes of Transmission. Handbook of Interpersonal Violence and Abuse Across the Lifespan2022. p. 2485-509.

8. McTavish JR, MacGregor JCD, Wathen CN, MacMillan HL. Children's exposure to intimate partner violence: an overview. International review of psychiatry (Abingdon, England). 2016;28(5):504-18.

9. Rikshandboken i barnhälsovård [Swedish national handbook for child health services]. Hälsobesök i alla åldrar: Inera AB; 2024 [Available from: https://www.rikshandboken-bhv.se/halsobesok/halsobesok-i-alla-aldrar/.

10. O'Doherty L, Hegarty K, Ramsay J, Davidson LL, Feder G, Taft A, et al. Screening women for intimate partner violence in healthcare settings. Cochrane database of systematic reviews. 2015;2015(8):CD007007-CD.

11. World Health Organization. Responding to Intimate Partner Violence and Sexual Violence against Women. WHO Clinical and policy guidelines. Geneva: World Health Organization; 2013. Report No.: 9789240691797.

12. Nimborg J, Lindskog U, Nordgren L, Annerbäck EM. Routine conversations about violence conducted in Swedish child health services—A mixed methods study of nurses' experiences. Acta Paediatrica. 2021;112(3):442-51.

For peer review only

For peer review only - http://bmjopen.bmj.com/site/about/guidelines.xhtml

13. Engström M, Lindqvist S, Janson S, Feldman I, Dubowitz H, Lucas S. Validation of the Swedish version of the safe environment for every kid (SEEK) parent screening questionnaire. BMC public health. 2023;23(1):1-1989.

14. Capaldi DM, Knoble NB, Shortt JW, Kim HK. A Systematic Review of Risk Factors for Intimate Partner Violence. Partner abuse. 2012;3(2).

15. Sundquist K, Malmström M, Johansson SE, Sundquist J. Care Need Index, a useful tool for the distribution of primary health care resources. Journal of epidemiology and community health (1979). 2003;57(5):347-52.

16. Damschroder LJ, Reardon CM, Widerquist MAO, Lowery J. The updated Consolidated Framework for Implementation Research based on user feedback. Implement Sci. 2022;17(1):75.

17. Anderzen-Carlsson A, Gillå C, Lind M, Almqvist K, Fändriks AL, Källström Å. Child healthcare nurses' experiences of asking new mothers about intimate partner violence. Journal of Clinical Nursing. 2018;27(13-14):2752-62.

18. O'Cathain A, Murphy E, Nicholl J. The quality of mixed methods studies in health services research. Journal of health services research & policy. 2008;13(2):92-8.

19. Fröjlinger A, Lalouni M, Blom L, Tiitinen Mekhail K, Lindberg L. Identification and response to family violence within Swedish Child Health Services: a pragmatic randomized controlled trial (manuscript submitted).

20. Braun V, Clarke V. Conceptual and Design Thinking for Thematic Analysis. Qualitative psychology (Washington, DC). 2022;9(1):3-26.

21. Fetters MD, Curry LA, Creswell JW. Achieving Integration in Mixed Methods Designs-Principles and Practices. Health services research. 2013;48(6pt2):2134-56.

For peer review only - http://bmjopen.bmj.com/site/about/guidelines.xhtml

Questionnaire for Child Health Centre Manager

- What is the name of the child health centre you manage?
- How many child health nurses were employed at the child health centre on July 1, 2024?
- How many child health nurses were employed at the child health centre on July 1, 2025?
- What percentage of the unit's child health nurses' working time is spent on child health services?
- How many child health nurses were employed during the period July 1, 2024 – July 1, 2025?
- How many child health nurses left during the period July 1, 2024 – July 1, 2025?
- For how many years have the unit's child health nurses worked in child health services?
- How many of the unit's child health nurses have participated in training on family violence?

For peer review only

Interview Guide: Asking About Violence in Child Health Services

Thank you for agreeing to participate in this interview. The aim of the study is to gain a deeper understanding of nurses' experiences of working with the identification of intimate partner violence within child health services. The focus is particularly on the experience of asking parents about violence.

Your responses will be treated confidentially and anonymized in the analysis and reporting. There are no right or wrong answers, I am interested in your personal experiences and reflections. The interview is expected to take approximately 30–60 minutes. You may withdraw from the interview at any time or skip any questions if you wish.

1. Characteristics of the intervention (the practice of asking about violence)

- How do you experience asking parents about violence within child health services?
- How clear do you find the guidelines regarding asking about violence?
- Do you think this way of working fits within your role as a child health nurse?
- What are your thoughts on asking both parents about violence?

2. Outer setting (external influences and support)

- How do parents usually respond when you ask about violence?
- Is there accessible support available for families who need it?
- How do you perceive collaboration with social services when making a report of concern?

3. Inner setting (organizational conditions)

- What kind of support is provided by management for working with issues related to violence at your workplace?
- Is there sufficient time and space during your meetings with parents to address questions about violence?
- What support is available at your workplace when making a report of concern in cases of suspected violence?

4. Characteristics of the individual (your own role and experience)

- How comfortable do you feel asking questions about violence?
- Have you received (sufficient) training, supervision, or other support to be able to talk about violence?
- Do you experience any differences in how you approach mothers compared to fathers when asking about violence?

For peer review only

- How do you feel about making a report of concern when you suspect that a child is being harmed?
- What do you think would make it easier for you to work with these issues?

5. Process (implementation of work with violence)

- How was the work of asking about violence introduced at your workplace?
- Are there opportunities within your team to discuss or reflect on these issues?
- How would you like the work with violence to develop in the future?

Closing

- Is there anything else you would like to add that we have not discussed?
- Is there anything you consider particularly important to highlight in this context?

For peer review only

BMJ Open

Response to family violence in child health services (FRIDa): study protocol of a mixed method study in the Stockholm region, Sweden

Journal:	*BMJ Open*
Manuscript ID	bmjopen-2025-115537.R2
Article Type:	Protocol
Date Submitted by the Author:	08-Jul-2026
Complete List of Authors:	Fröjlinger, Anna; Karolinska Institutet, Department of Global Public Health<br>Blom, Lisa; Karolinska Institute, Department of Global Public Health<br>Tiitinen Mekhail, Kirsi; Department of Neurobiology, Care Sciences and Society<br>Lalouni, Maria; Karolinska Institute, Dept of Clinical Neuroscience; Region Stockholm, Center for Epidemiology and Community Medicine<br>Lindberg, Lene; Karolinska Institutet, Department of Global Public Health; Region Stockholm, Center for Epidemiology and Community Medicine
**Primary Subject Heading**:	Health services research
Secondary Subject Heading:	Public health, Paediatrics
Keywords:	Gender-Based Violence, Child protection < PAEDIATRICS, Nursing research, Community child health < PAEDIATRICS

SCHOLARONE
MANUSCRIPTS

Response to family violence in child health services (FRIDa): study protocol of a mixed method study in the Stockholm region, Sweden

Anna Fröjlinger (0009-0006-5411-6633)[1], Lisa Blom (0000-0002-3538-7159)[1], Kirsi Tiitinen Mekhail (0000-0002-5715-8656)[2], Maria Lalouni (0000-0002-6818-8156)[3,4], Lene Lindberg (0000-0002-9275-551X)[1,4]

[1]Department of Global Public Health, Karolinska Institutet, Solnavägen 1E, 113 65 Stockholm, Sweden

[2] Department of Neurobiology, Care Sciences and Society, Alfred Nobels allé 23, 141 52 Huddinge, Sweden

[3] Department of Clinical Neuroscience, Karolinska Institutet, Tomtebodavägen 18A, 171 77 Stockholm, Sweden

[4] Center for Epidemiology and Community Medicine, Region Stockholm, Solnavägen 1E, 113 65 Stockholm, Sweden

Correspondence to: A Fröjlinger anna.frojlinger@ki.se

Word count (main text only): 3328

For peer review only

For peer review only - http://bmjopen.bmj.com/site/about/guidelines.xhtml

Abstract

Introduction: Growing up with family violence (FV) is potentially harmful both in a short- and long-term perspective for the child. Early identification and support are therefore important. The purpose of this project (FRIDa) is to gain a deeper knowledge about identification of and response to FV in the Swedish Child Health Services (CHS). Further, we would like to better understand how structure and organization together with nurses' perceptions may contribute to identification and response within the CHS.

Methods and analysis: In this part of the FRIDa-project, we will use a mixed method design to explore the pattern of identification of, and response to, FV. Approximately 90 000 CHS medical records over 12 months will be analyzed with regression analysis to examine how FV identification and response vary by demographic factors. Furthermore, a qualitative interview study will gain information about the organizational barriers and facilitators, and nurses' professional experiences of working with FV within the CHS. The quantitative and qualitative results will be integrated in an explorative data analysis focusing on variations in the frequencies of posed questions, identification and response to FV supported by interview data.

Ethics and dissemination: The study was approved by the Swedish Ethical Review Authority (Reference number 2025-03516-01). Results will be published with open access in international peer-review journals and communicated to national and regional CHS to contribute to the understanding of the long-term clinical work with FV within CHS. It will also add to the advancement of evidence-based practices for identification of, and response to, FV.

Trial registration: ClinicalTrials.gov NCT07158983. Registered on August 28, 2025.

For peer review only

Strengths and limitations of this study

- A mixed-methods design integrates quantitative register data and qualitative interview data.
- The large register dataset of approximately 90,000 child health records provides high statistical power for quantitative analyses.
- The inclusion of two major child health service providers increases variation in organisational and socioeconomic contexts.
- The study is conducted within a single Swedish region, which may limit transferability to other regions and countries.
- Voluntary participation in the interview study may introduce selection bias among participating nurses.

Keywords

Family Violence, Child Health Services, Identification, Support, Mixed Methods, Parental Support

For peer review only

Introduction

Family violence (FV) is a serious global public health concern [1]. The prevalence of FV varies depending on definition, how, when, and where data has been collected [2,3]. Children may experience FV through direct exposure (e.g. physical, psychological or sexual abuse), or by witnessing violence between caregivers or other family members. While these forms of exposure often co-occur, they represent conceptually distinct experiences [4]. Globally, a large proportion of children are exposed to FV [2]. In Sweden, self-reported data from adolescents regarding experiences during childhood indicate that 12 and 13% have been subjected to psychological or physical abuse by a caregiver, respectively, and around 10% have witnessed violence between adults in the home [5]. Short- and long-term consequences for children experiencing FV have been described concerning physical health, cognitive, emotional and behavioral problems or disorders [6]. Another identified consequence associated with exposure to FV during childhood is the risk of being a victim or perpetrator of FV in adulthood [7]. The consequences may be similar whether the child has witnessed violence between adults or has been directly exposed to violence [8].

Early identification of FV within child health services (CHS) is considered an important strategy for mitigating negative effects for children. In Sweden, CHS is a nurse-led, universal service offered free of charge to all children aged 0–5 years and their parents, reaching almost all families. All parents are offered an individual consultation with a CHS nurse during the child's first six months, providing an important opportunity to ask about exposure to FV. The high coverage and repeated contacts over time make CHS a central arena for identifying FV [9].

Whether detection of FV should be administered through questions on routine or by indication is not evident. Research so far has failed to demonstrate that routine questions

For peer review only

For peer review only - http://bmjopen.bmj.com/site/about/guidelines.xhtml

about FV reduce the prevalence of FV [10]. That may, however, be explained by lack of effective interventions, rather than by the identification process. WHO recommends that services with regular contact with women and children consider routine enquiry about FV, whereas services with less frequent contact may instead use targeted questions based on identified risk factors [11]

Few studies have examined how questions on exposure to violence posed to new fathers in the healthcare settings are working. A few Swedish studies from the CHS context report a lower share of fathers than mothers being asked about violence [12,13]. To our knowledge, no international studies have investigated how fathers are offered the opportunity to answer questions about their own exposure to violence within a child health or parenting support context.

FV exists in all societies and cultures but there is an increased risk in areas with a higher degree of social vulnerability [14]. Examining whether parents in areas with different socioeconomic conditions are equally likely to be asked about FV is therefore important. Social vulnerability at a societal level can be measured in several ways. One such measure used within CHS is Care Need Index (CNI), a socioeconomic index used to estimate healthcare needs within an area [15]. It is based on factors such as single parenthood, unemployment, low educational level, and proportion of foreign-born residents in the area. A higher CNI indicates a greater need for support and resources in primary health care.

Maintaining routines for asking about violence over time can be difficult, as these practices are often influenced by factors such as workload, staff turnover, and access to training. Even routines that are initially well implemented may diminish or become less consistently applied over time [16]. These organizational factors may also influence healthcare

For peer review only

For peer review only - http://bmjopen.bmj.com/site/about/guidelines.xhtml

professionals´ confidence in addressing violence, including their preparedness to act when violence is identified. Previous research suggests that a prerequisite for healthcare professionals to ask about exposure to violence is to feel confident about actions and steps to follow if violence is identified [17]. In Swedish CHS, existing research has focused on the implementation phase of such routines [12,13,17]. Less is known about how these practices are sustained over time and how nurses experience working with them in everyday clinical practice.

The present study addresses this gap by examining the experiences of CHS nurses who have worked with this approach for several years, providing insights into long-term practice rather than initial adoption. Furthermore, the approach in this study includes asking both mothers and fathers in the same family about their own exposure to violence, which is uncommon in previous research. This perspective is important for understanding how routine questions can support violence prevention and parenting support in a more inclusive way.

To sum up, exposure to FV among children is a severe public health problem that could be reduced by early identification and response in CHS. More research is needed to examine the opportunities of mothers and fathers, and parents in different socioeconomic areas to be asked about violence during CHS encounters. There is also a need for increased knowledge on the support CHS nurses require to ask about violence and respond when violence is identified, particularly from a long-term perspective.

Methods and analysis

Aim

The aim of this project is to gain a deeper knowledge on how FV is identified and

For peer review only - http://bmjopen.bmj.com/site/about/guidelines.xhtml

responses to FV within the Swedish CHS. Further, we aim to understand how structure and organization together with nurses' perceptions may contribute to identification of and response to FV within the CHS.

Research questions

The specific research questions are:

- To what extent are questions about FV posed to mothers and fathers, respectively, in CHS?
- To what extent is ongoing FV identified among parents that have been asked about it?
- To what extent have reports of concern been made to social services when FV is identified?
- To what extent are structural factors (e.g., CNI) and organizational conditions (e.g. professional education and supervision) associated with nurses' likelihood of inquiring about FV and responding when FV is identified within CHS?"
- What barriers and facilitators do nurses within CHS perceive when asking about FV?
- What barriers and facilitators do nurses within CHS perceive in providing support to families affected by identified FV?

Design

The study will use a mixed method design including both quantitative and qualitative approaches. In the quantitative part of the study, data from CHS medical records will be used to identify the frequency of questions posed on routine and based on indication and reports of FV within the CHS. Data from clinical managers are used to map the

For peer review only - http://bmjopen.bmj.com/site/about/guidelines.xhtml

organizational preconditions of the CHS clinics. The qualitative part of the study consists of interviews with nurses to explore their experiences of identifying and responding to identified FV. The combination of methods will support the understanding of the situation around working with identifying FV within CHS. The study protocol was developed according to the Good Reporting of a Mixed Methods Study (GRAMMS). GRAMMS is used as a checklist to ensure that relevant information is included in the study protocol and it will also be used in the reporting of the results [18].

Study setting

The study is set in the Stockholm region in Sweden and involve two of the largest caregivers providing CHS in Stockholm region - Stockholm Health Care Services and Capio. Together they account for about half of the children enrolled at CHS clinics in the region. The Stockholm region comprises about 20% of all children residing in Sweden. FRIDa is a research project aimed at supporting CHS in identifying and responding to FV through structured routines and staff training [19]. Based on the FRIDa model, routine questions about FV have been implemented in the CHS in the Stockholm region. For the mother, this usually takes place during an individual visit when the child is 6-8 weeks and for the father at an individual visit when the child is 3-5 months. Three questions are asked, relating to 1) prior exposure to FV for the parent, 2) ongoing exposure to FV for the parent, and 3) ongoing exposure to FV for the child. Questions on exposure to violence should also be asked at any time based on indication, whenever there is a suspicion of violence.

For peer review only

Quantitative part

Objectives

The objective of the quantitative part of the study is to examine how questions about FV are asked within CHS in the Stockholm region, and how healthcare professionals respond when violence is identified. A further objective is to examine how these practices are associated with organizational and contextual factors at the CHS clinic level.

Data sources

The quantitative part will draw on two complementary data sources:

1. ***Registry-based data from CHS medical records***

Participants

We will make use of registry data from medical records of children enrolled in the CHS during the period 2024-06-30 to 2025-06-30. Approximately 90 000 children are enrolled in the CHS provided by the two included health care providers. The included CHS clinics are based in areas with different socioeconomic characteristics (according to CNI) located in the city, suburbs and surrounding municipalities in the Stockholm region.

Measurements and data collection

De-identified medical records from CHS will be compiled with support from administrators at the two care providers. There are specific variable names in the medical records for questions about exposure to violence, reports of concern to social services, and whether the mother or father had an individual visit with the CHS nurse.

The data that will be retrieved from the CHS medical records are: date for the visit; age of the child; type of contact; information about whether an individual visit has taken place for the parent (yes/no); whether a question on exposure to violence has been asked (yes/no)

For peer review only - http://bmjopen.bmj.com/site/about/guidelines.xhtml

and the parents' answer; notes about exposure to violence for the child (yes/no); reports of concern to social services (yes/no); and reason for reports of concern to social services (when a report is made).

Since questions about violence should be asked whenever there is a suspicion of violence, all medical records from the CHS of the two care providers are eligible for inclusion in the data collection. The total number of enrolled children will be used as the denominator when calculating frequencies. Information on FV assessment will be extracted from medical records where documentation of 1) an individual parental consultation, 2) questions about violence, or 3) a report of concern is present during the study period.

2. *Organizational characteristics of CHS clinics*

Sampling and recruitment

All unit managers and operations managers at the participating CHS clinics will be contacted.

Data collection

An electronic questionnaire with questions about organizational characteristics of the included CHS clinics will be distributed via a secure web platform (see supplementary file 1). The questions cover information on the number of nurses employed, their percentage of full-time employment, years of experience in CHS, number of enrolled children and how many of the nurses that have participated in training about FV. The questionnaire also includes questions on staff turnover in the latest year. It is estimated to take about 15 minutes to complete the questionnaire. The CNI for each CHS clinic is publicly available

For peer review only

For peer review only - http://bmjopen.bmj.com/site/about/guidelines.xhtml

and will be retrieved from Region Stockholm.

Informed consent

An information letter will be distributed to all unit managers and operations managers of the included CHS clinics. The information letter includes information about the voluntariness to complete the questionnaire, that all information will be reported on a group level without possibilities to identify specific CHS clinics or nurses and that the data collected will only be available to the researchers involved and stored securely at Karolinska Institutet.

Data integration

Data from medical records on FV assessment will be linked to corresponding clinic-level variables, including CNI and organizational characteristics, to examine how contextual factors may be associated with assessment practices.

Analysis

Descriptive statistics will be used to calculate the frequency of 1) questions about FV posed to parents, 2) identification of ongoing violence, and 3) reports of concern following identification. Differences between mothers and fathers in being asked about violence will be examined using appropriate statistical tests depending on the data distribution. Regression analyses will be conducted to explore associations between questions about violence, CNI and organizational and clinic-level variables. All analyses will be conducted using the statistical software IBM SPSS Statistics for Windows, Version 31.0 (IBM Corp., Armonk, NY, USA).

For peer review only - http://bmjopen.bmj.com/site/about/guidelines.xhtml

The qualitative part

Objectives

The objective of the qualitative part of the study is to gain insight into nurses' perspectives on, and experiences from, working with questions about FV in CHS and how it is embedded and sustained in the routine practice. An additional objective is to better understand how they respond when FV is identified.

Sampling and recruitment

Based on the results of the quantitative part, a selection of 10-15 nurses will be approached and asked for participation in semi-structured individual interviews. The selection will consist of nurses working at CHS clinics with different CNI and with high and low frequencies of parents being asked about FV according to the results from the quantitative part.

Data collection

Semi-structured individual interviews will be conducted focusing on barriers and facilitating factors to pose questions on exposure to violence and to respond when violence exposure is identified. The interview guide (see Supplementary file 2) is informed by the Consolidated Framework for Implementation Research (CFIR) that can be used to support the understanding of factors affecting the implementation of evidence-based methods and interventions [16]. The guide includes questions addressing experiences of asking about violence, perceived clarity of guidelines, organizational support and conditions, collaboration with external actors, as well as individual confidence and preparedness to ask about and respond to violence. The interviews will be conducted face-to-face or digitally via Zoom or Teams after informed consent has been signed. The interviews will be

For peer review only

audio-recorded and thereafter transcribed verbatim.

Informed consent

Written informed consent will be obtained from the nurses prior to participating in the interviews. The nurses will be informed that participation is voluntary and that data will be presented in a way minimizing the risk of identifying individual participants. Nurses participating in the interviews will also have access to support from a psychologist from the study team.

Analysis

The interviews will be analyzed using reflexive thematic analysis [20]. The analysis will involve an iterative and recursive process, moving back and forth between reading the transcripts, coding the data, and developing themes. The researchers will read the transcripts and contribute to the analytic process through ongoing discussion. The focus of the analysis will be on exploring variations in nurses' experiences of asking about, identifying, and responding to FV within CHS.

Integration of the results from the quantitative and the qualitative parts

Results from the two parts will be integrated in an explorative data analysis with a mixed method approach [21]. The focus on the analysis will be to explore variations in the frequency of questions posed, identification and response to FV supported by interview data.

Study timeline

Ethical approval was sought and granted in May 2025. The care givers were approached

For peer review only - http://bmjopen.bmj.com/site/about/guidelines.xhtml

and requested for patient- and organizational data during October/November 2025. Data management and analysis of the register data was initiated in February 2026 and will be finalized in November 2026. Nurses at selected CHS clinics will be contacted with written information and invitation to participate in interviews in January 2027. Interviews are planned to be conducted in March-June 2027. Data management and analysis of the interview data will be conducted in March-December 2027. The integration of the results of the material from the registry review and the interviews will be compiled and presented in January-June 2028. The timeline of the study is described in Supplementary file 3 Table 1.

Ethics and dissemination

The study was approved by the Swedish Ethical Review Authority (Reference number 2025-03516-01). Nurses will provide written informed consent before participating in the interviews. All results will be presented at a group level. The final data set will be handled in accordance with the guidelines of Karolinska Institutet. The variable list and the information letters are available from the corresponding author on request.

The results of the study will be published with open access in peer-reviewed journals and presented to regional and national CHS.

Patient and public involvement

Patients and the public were not involved in the design, conduct, reporting, or dissemination plans of this research. However, the results will be shared with CHS staff to inform practice.

Discussion

For peer review only - http://bmjopen.bmj.com/site/about/guidelines.xhtml

This protocol describes a mixed methods study designed to gain a deeper knowledge on identification of, and response to, FV within the Swedish CHS. Considering the serious health consequences that violence can cause, it is important that healthcare introduces evidence-based methods that facilitate identification and ensure equitable opportunities for support for victims of violence.

There are several practical and operational issues to discuss and reflect on in the upcoming study. One relates to the two outcomes identification of exposure to violence for and reports of concern to social services. Both outcomes are rare events which can make comparisons difficult, as reported in a previous study within the research group [19]. The large material in this study is expected to cater for better comparisons.

Another issue concerns the selection of CHS clinics. Only two care providers of CHS are included due to practical and logistical reasons. However, the two care providers together account for a large share of the CHS clinics in the uptake area and include clinics located in areas with varying socioeconomic conditions and are expected to provide a representative sample of the CHS clinics in the region.

Several considerations related to the selection of nurses and the interviews should also be lifted. The selection of nurses is made with the purpose of providing information from a variation of CHS clinics with different CNI and with low and high frequencies of parents that have been asked about exposure to violence. The nurses are selected based on the level of the CHS clinic instead of the individual nurses' levels, to decrease the risk of nurses feeling exposed. A downside of that is that we will not be able to control the selection of the individual nurses at a specific clinic, where nurses with a more positive perspective on asking questions around violence might be more willing to participate in the interviews. To mitigate this, the study emphasizes that interviews focus on barriers and facilitators and that confidentiality will be strictly maintained. Another concern is the choice

For peer review only - http://bmjopen.bmj.com/site/about/guidelines.xhtml

of interviewer which may affect how comfortable nurses feel about sharing their experiences and perspectives. We will strive to select an interviewer that has no, or limited, connection to the day-to-day work of the nurses.

The study is expected to support the possibilities for children's and parents' equal opportunities for support via the CHS in case of FV. This is expected to be achieved by increasing knowledge about potential differences in how questions on violence are posed to parents related to gender, area level socioeconomic background and different possibilities, both actual preconditions and experienced opportunities, for nurses to comply with the method implemented. A strength of the study is its focus on nurses who have worked with the method for several years, offering insights into sustainability and normalization of the approach beyond the initial implementation phase. This long-term perspective is important for understanding not only feasibility but also how the method becomes embedded in routine practice. The findings may inform strategies for organizational support and training, ensuring that CHS nurses have the necessary conditions to maintain this practice over time. This could strengthen the capacity of CHS to identify FV early and provide equal opportunities for support to all parents, contributing to violence prevention and safer environments for children.

Abbreviations

CHS – Child health services

CNI – Care need index

EPDS – Edinburgh Postnatal Depression Scale

FV – Family violence

For peer review only

For peer review only - http://bmjopen.bmj.com/site/about/guidelines.xhtml

Author contributions

AF and LB drafted the manuscript. LL is the principal investigator, and she conceived the study together with AF. All authors (AF, LB, KTM, ML and LL) have contributed to the study design and have critically revised the draft, read and approved the final manuscript.

Guarantor: LL

Funding

This work was supported by the Swedish Research Council for Health, Working life and Welfare (Forte), reference number: 2023-01144. Open access funding was provided by Karolinska Institutet. The funders had no role in the design of the study and will have no role in the data collection, analysis and interpretation or in writing the manuscript.

Competing interests

The authors declare that they have no competing interests.

References

1. Krug EG, Mercy JA, Dahlberg LL, Zwi AB. The world report on violence and health. Geneva; 2002.

2. UNICEF. A Familiar Face: Violence in the lives of children and adolescents. New York; 2017.

3. World Health Organization. Violence against women prevalence estimates, 2018: global, regional and national prevalence estimates for intimate partner violence against women and global and regional prevalence estimates for non-partner sexual violence against women. Geneva: World Health Organization; 2021.

For peer review only

For peer review only - http://bmjopen.bmj.com/site/about/guidelines.xhtml

4. Howell KH, Taylor NR, Thomsen KN, Martinez-Torteya C, Källström Å. Global Perspectives on Family Violence: Prevalence and Effects on Children across Cultural Contexts. Psychological Perspectives on Understanding and Addressing Violence Against Children2022.

5. Jernbro C, Landberg Å, Thulin J. Våld mot barn 2022: En nationell kartläggning. Stockholm; 2023. Report No.: 91-86759-57-4978-91-86759-57-5.

6. Howell KH, Barnes SE, Miller LE, Graham-Bermann SA. Developmental variations in the impact of intimate partner violence exposure during childhood. Journal of injury and violence research. 2016;8(1):43-57.

7. Ehrensaft MK, Langhinrichsen-Rohling J. Intergenerational Transmission of Intimate Partner Violence: Summary and Current Research on Processes of Transmission. Handbook of Interpersonal Violence and Abuse Across the Lifespan2022. p. 2485-509.

8. McTavish JR, MacGregor JCD, Wathen CN, MacMillan HL. Children's exposure to intimate partner violence: an overview. International review of psychiatry (Abingdon, England). 2016;28(5):504-18.

9. Rikshandboken i barnhälsovård [Swedish national handbook for child health services]. Hälsobesök i alla åldrar: Inera AB; 2024 [Available from: https://www.rikshandboken-bhv.se/halsobesok/halsobesok-i-alla-aldrar/.

10. O'Doherty L, Hegarty K, Ramsay J, Davidson LL, Feder G, Taft A, et al. Screening women for intimate partner violence in healthcare settings. Cochrane database of systematic reviews. 2015;2015(8):CD007007-CD.

11. World Health Organization. Responding to Intimate Partner Violence and Sexual Violence against Women. WHO Clinical and policy guidelines. Geneva: World Health Organization; 2013. Report No.: 9789240691797.

For peer review only - http://bmjopen.bmj.com/site/about/guidelines.xhtml

12. Nimborg J, Lindskog U, Nordgren L, Annerbäck EM. Routine conversations about violence conducted in Swedish child health services—A mixed methods study of nurses' experiences. Acta Paediatrica. 2021;112(3):442-51.

13. Engström M, Lindqvist S, Janson S, Feldman I, Dubowitz H, Lucas S. Validation of the Swedish version of the safe environment for every kid (SEEK) parent screening questionnaire. BMC public health. 2023;23(1):1-1989.

14. Capaldi DM, Knoble NB, Shortt JW, Kim HK. A Systematic Review of Risk Factors for Intimate Partner Violence. Partner abuse. 2012;3(2).

15. Sundquist K, Malmström M, Johansson SE, Sundquist J. Care Need Index, a useful tool for the distribution of primary health care resources. Journal of epidemiology and community health (1979). 2003;57(5):347-52.

16. Damschroder LJ, Reardon CM, Widerquist MAO, Lowery J. The updated Consolidated Framework for Implementation Research based on user feedback. Implement Sci. 2022;17(1):75.

17. Anderzen-Carlsson A, Gillå C, Lind M, Almqvist K, Fändriks AL, Källström Å. Child healthcare nurses' experiences of asking new mothers about intimate partner violence. Journal of Clinical Nursing. 2018;27(13-14):2752-62.

18. O'Cathain A, Murphy E, Nicholl J. The quality of mixed methods studies in health services research. Journal of health services research & policy. 2008;13(2):92-8.

19. Fröjlinger A, Lalouni M, Blom L, Tiitinen Mekhail K, Lindberg L. Identification and response to family violence within Swedish Child Health Services: a pragmatic randomized controlled trial (manuscript submitted).

20. Braun V, Clarke V. Conceptual and Design Thinking for Thematic Analysis. Qualitative psychology (Washington, DC). 2022;9(1):3-26.

For peer review only

For peer review only - http://bmjopen.bmj.com/site/about/guidelines.xhtml

21. Fetters MD, Curry LA, Creswell JW. Achieving Integration in Mixed Methods Designs-Principles and Practices. Health services research. 2013;48(6pt2):2134-56.

For peer review only

For peer review only - http://bmjopen.bmj.com/site/about/guidelines.xhtml

Questionnaire for Child Health Centre Manager

- What is the name of the child health centre you manage?
- How many child health nurses were employed at the child health centre on July 1, 2024?
- How many child health nurses were employed at the child health centre on July 1, 2025?
- What percentage of the unit's child health nurses' working time is spent on child health services?
- How many child health nurses were employed during the period July 1, 2024 – July 1, 2025?
- How many child health nurses left during the period July 1, 2024 – July 1, 2025?
- For how many years have the unit's child health nurses worked in child health services?
- How many of the unit's child health nurses have participated in training on family violence?

For peer review only

Interview Guide: Asking About Violence in Child Health Services

Thank you for agreeing to participate in this interview. The aim of the study is to gain a deeper understanding of nurses' experiences of working with the identification of intimate partner violence within child health services. The focus is particularly on the experience of asking parents about violence.

Your responses will be treated confidentially and anonymized in the analysis and reporting. There are no right or wrong answers, I am interested in your personal experiences and reflections. The interview is expected to take approximately 30–60 minutes. You may withdraw from the interview at any time or skip any questions if you wish.

1. Characteristics of the intervention (the practice of asking about violence)

- How do you experience asking parents about violence within child health services?
- How clear do you find the guidelines regarding asking about violence?
- Do you think this way of working fits within your role as a child health nurse?
- What are your thoughts on asking both parents about violence?

2. Outer setting (external influences and support)

- How do parents usually respond when you ask about violence?
- Is there accessible support available for families who need it?
- How do you perceive collaboration with social services when making a report of concern?

3. Inner setting (organizational conditions)

- What kind of support is provided by management for working with issues related to violence at your workplace?
- Is there sufficient time and space during your meetings with parents to address questions about violence?
- What support is available at your workplace when making a report of concern in cases of suspected violence?

4. Characteristics of the individual (your own role and experience)

- How comfortable do you feel asking questions about violence?
- Have you received (sufficient) training, supervision, or other support to be able to talk about violence?
- Do you experience any differences in how you approach mothers compared to fathers when asking about violence?

For peer review only

- How do you feel about making a report of concern when you suspect that a child is being harmed?
- What do you think would make it easier for you to work with these issues?

5. Process (implementation of work with violence)

- How was the work of asking about violence introduced at your workplace?
- Are there opportunities within your team to discuss or reflect on these issues?
- How would you like the work with violence to develop in the future?

Closing

- Is there anything else you would like to add that we have not discussed?
- Is there anything you consider particularly important to highlight in this context?

For peer review only

Table 1. Timeline of the study

	2025		2026				2027				2028	
**Timepoint**	**Q3**	**Q4**	**Q1**	**Q2**	**Q3**	**Q4**	**Q1**	**Q2**	**Q3**	**Q4**	**Q1**	**Q2**
**Quantitative part: Register data and CHS clinic organizational data**												
Ethical approval	X											
Data collection		X	X									
Data management & analysis			X	X	X	X						
**Qualitative part: interview data**												
Ethical approval	X											
Recruitment							X					
Interviews							X	X				
Data management & analysis								X	X	X		
**Integration of the results of the quantitative and qualitative parts**												
Compilation & presentation of results											X	X

For peer review only
